# Building resilience against the growing threat of arboviruses: a scoping review of *Aedes* vector surveillance, control strategies and insecticide resistance in Africa

**DOI:** 10.1186/s13071-025-07049-7

**Published:** 2025-10-17

**Authors:** Richard M. Oxborough, Basiliana Emidi, Aurelie P. Yougang, Tarekegn A. Abeku, Fatima Ahmed, Joseph R. Biggs, Kallista Chan, Jackie Cook, Amy Edwards, Jane Falconer, Basile Kamgang, Louisa A. Messenger, Frederik Seelig, Roz Taylor, Armel N. Tedjou, Jo Lines, Sian E. Clarke, Mojca Kristan

**Affiliations:** 1 Parasitology and Vector Biology (PARAVEC) Laboratory, School of Public Health, University of Nevada, Las Vegas, NV USA; 2 Department of Biological Sciences, College of Southern Nevada, Las Vegas, NV USA; 3 National Institute for Medical Research, Dodoma Centre, P. O. Box 805, Dodoma, Tanzania; 4 Centre for Research in Infectious Diseases (CRID), P.O. Box 13591, Yaoundé, Cameroon; 5 Malaria Consortium, London, UK; 6 Department of Disease Control, Faculty of Infectious and Tropical Diseases, London School of Hygiene and Tropical Medicine, London, WC1E 7HT UK; 7 International Statistics and Epidemiology Group, Department of Infectious Disease Epidemiology and International Health, Faculty of Epidemiology and Population Health, London School of Hygiene and Tropical Medicine, London, WC1E 7HT UK; 8 User Support Services Librarian, Library, Archive & Open Research Services, London School of Hygiene & Tropical Medicine, London, UK; 9 Aarhus University, Aarhus, Denmark

**Keywords:** *Aedes aegypti*, *Aedes albopictus*, Arbovirus, Chikungunya, Dengue, Yellow fever, Zika, Insecticide resistance, Vector control, Surveillance

## Abstract

**Background:**

The number of reports of arboviral outbreaks is increasing and, consequently, the need for effective surveillance and vector control plans for *Aedes*-borne diseases is becoming more urgent. To explore the current state of knowledge of *Aedes* arbovirus vectors in Africa, we reviewed studies published between 1980 and 2023 that involved *Aedes* vector surveillance, vector control or insecticide resistance, with the aim to synthesize information and identify knowledge gaps to guide future *Aedes* research and control in Africa.

**Methods:**

Studies conducted in Africa and published between 1980 and 2023 were retrieved from twelve electronic databases using search strings designed to capture relevant concepts. Articles that did not meet the eligibility criteria were excluded during relevance screening.

**Results:**

Out of 17,337 publications identified, 877 full-text articles were reviewed, of which seven included information on vector surveillance, 56 on vector control and 57 on insecticide resistance. Publications reporting longitudinal data from sustained *Aedes* vector surveillance systems were only available for Senegal and La Réunion. *Aedes* vector control studies were principally controlled bioassays or small-scale studies conducted before and after entomological studies which lacked epidemiological outcomes. The most studied methods were larval control (*n* = 21 publications), integrated control combining different interventions (*n* = 7), topical repellents (*n* = 6), environmental management (*n* = 5) and spatial repellents (*n* = 3). Four publications described typical vector control responses during arbovirus epidemics in Africa: these often combined larviciding, ultra-low volume (ULV) space spraying and community engagement to reduce larval sites, alongside active source reduction. There was a lack of high-quality evidence generated through rigorous study design on the effectiveness of control measures in reducing arbovirus transmission in the African context. As a consequence, the scientific basis for evidence-informed decisions in Africa, both for routine *Aedes* vector control or for outbreak response, remains weak. Insecticide resistance studies focused on adulticides using WHO tube tests (*n* = 43 publications), with larval bioassays relatively less common (*n* = 13). *Aedes aegypti* (*n* = 53) and *Aedes albopictus* (*n* = 12) were the only *Aedes* species tested. The most commonly tested adulticides were permethrin and deltamethrin (pyrethroids); bendiocarb (carbamate); and dichlorodiphenyltrichloroethane (DDT; organochlorine), although the results were rarely reported in connection with decision-making about *Aedes* control. Results of the most relevant adulticides indicated that *Ae. aegypti* populations were generally susceptible to malathion (organophosphate), but resistance to permethrin and deltamethrin was detected in West and Central Africa. Most studies pre-dated the revised WHO guidance, and insecticide concentrations were mostly those recommended for *Anopheles* susceptibility testing that use relatively higher discriminating doses, and thus likely underestimate true *Aedes* resistance levels. Larval susceptibility bioassays were conducted with temephos (*n* = 12) and *Bacillus thuringiensis israelensis* (*n* = 6). Temephos resistance was only detected in Cabo Verde following several decades of use.

**Conclusions:**

Given the increasing frequency of arbovirus epidemics in Africa, countries urgently need to develop plans for emergency response and robust control strategies that make use of evidence from good-quality studies to strengthen resilience.

**Supplementary Information:**

The online version contains supplementary material available at 10.1186/s13071-025-07049-7.

## Background

*Aedes* mosquitoes transmit several arboviruses, including yellow fever, dengue, chikungunya and Zika, all of which pose significant public health threats across Africa. However, despite the health risks posed by these arboviruses, the distribution and disease burden of arboviruses in Africa is understudied, mosquito control efforts are predominantly malaria-oriented and health services are often under-resourced, making it difficult to effectively detect and manage arboviral diseases [1]. Many arboviruses have origins in Africa [2–4], as does the mosquito *Aedes aegypti* [5, 6], which remains a primary vector of arboviruses around the world. The second most important arboviral vector, *Aedes albopictus*, was introduced into mainland Africa from Asia in the early 1990s and has since spread across the continent [7, 8]. In addition, over 15 *Aedes* species native to Africa are competent arbovirus vectors, including *Ae. furcifer*, *Ae. luteocephalus*, and *Ae. simpsoni* complex, amongst others [9, 10].

Entomological surveillance and vector control in Africa have historically been malaria-centric [11]. The burden of malaria has decreased in many parts of Africa since 2000–mainly due to the widespread use of interventions, such as insecticide-treated nets (ITNs) and to a lesser extent indoor residual spraying (IRS) [12]–a development that has led to arboviral infections and haemorrhagic fevers becoming increasingly recognized as public health threats [13–15]. The largest ever dengue outbreak in the region occurred in 2023 and accounted for 80% of all-time cases and 92% of fatalities, with Burkina Faso particularly affected (75% of cases and 91% of fatalities) [16, 17]. As arboviral outbreaks are increasingly reported, the need for effective surveillance and epidemic response plans for *Aedes*-borne diseases becomes more urgent. This is particularly important in Africa, where the situation could worsen due to factors such as rapid urbanization and climate change, which are likely to expand the geographic range and abundance of *Aedes* mosquitoes [18–20]. Growing urban populations have long been associated with a reduced malaria burden, in part due to physical landscape modifications that are unfavourable to malaria vectors [21]. In contrast, *Ae. aegypti* and *Ae. albopictus* are well adapted to urban environments, where they breed in artificial containers that collect water, such as buckets, tyres and discarded plastic [22].

We conducted a scoping review of all literature published between 1980 and 2023 to explore the state of knowledge of *Aedes* arbovirus vectors in Africa, specifically regarding vector control strategies, insecticide resistance and entomological surveillance. Through a synthesis of this information and identification of major knowledge gaps, we aimed to develop recommendations to guide future research to counter the problem of expanding and frequently occurring outbreaks of arboviral diseases in Africa. This scoping review can be used to assist national disease control programmes and other stakeholders to support the development of effective vector surveillance programmes and control strategies.

## Methods

A scoping review protocol was created and is published through the Open Science Foundation (OSF) (10.17605/OSF.IO/VZ5GH) [23]. This scoping review was designed according to the six stages of the scoping review process outlined by Arksey and O’Malley [24, 25], namely (i) identifying research questions; (ii) identifying relevant studies; (iii) screening of studies; (iv) charting the data; (v) collating and summarizing results from extracted data; and (vi) consultation with stakeholders, including national ministries of health. The present study is reported according to PRISMA-ScR (Preferred Reporting Items for Systematic Reviews and Meta-Analyses Extension for Scoping Reviews) guidelines [26]. We initially conducted a preliminary, exploratory review of some of the available recent literature to develop a list of more detailed study questions (sub-questions) which were then categorized to produce two scoping reviews. Sub-questions for this review (which is focused on entomological surveillance systems, vector control and outbreak response) included determining the primary methods of *Aedes* vector control in Africa and evidence for their effectiveness; effects of malaria vector control interventions on *Aedes* vector populations; and observations that indicate the role of vector control in the prevention and control of arboviral outbreaks. We determined the state of insecticide resistance monitoring of *Aedes* vectors in Africa, including bioassay methodologies, insecticides, concentrations and mosquito species used and major mechanisms of insecticide resistance. Vector surveillance focused on longitudinal studies that involved routine, systematic monitoring of *Aedes* populations over a prolonged period (multiple years) to inform public health interventions.

### Search strategy

Searches were conducted in 12 electronic databases that index published peer-reviewed and grey literature. The search included strings of terms, synonyms and controlled vocabulary terms (where available) to reflect three concepts:

- Concept 1: *Aedes* mosquitoes and the diseases and pathogens they spread
- Concept 2: Surveillance, vector control, insecticide resistance, *Aedes* distribution, bionomics and vector competence
- Concept 3: Africa.

The complete strings of search terms are published through the London School of Hygiene and Tropical Medicine (LSHTM) Data Compass digital repository (10.17037/DATA.00004717) [27]. Databases were identified based on their relevance to the topic following a consultation with an experienced librarian/information specialist who performed the searches. The following bibliographic databases were searched in January 2023: EBSCOhost Africa-Wide Information, Wiley Cochrane Central Register of Controlled Trials, OvidSP Embase + Embase Classic, OvidSP Global Health, OvidSP Medline ALL, Clarivate Analytics Web of Science, BIOSIS Citation Index, Clarivate Analytics Web of Science Core Collection, Science Citation Index Expanded (SCI-Expanded), Social Sciences Citation Index (SSCI), Arts & Humanities Citation Index (A&HCI), Conference Proceedings Citation Index-Science (CPCI-S), Conference Proceedings Citation Index-Social Science & Humanities (CPCI-SSH) and Emerging Sources Citation Index (ESCI). The following clinical trials registers were also searched in January 2023: Clinical Trials.gov (complete database) and WHO International Clinical Trials Registry Platform (ICTRP). Search strategy verification was done by checking reference lists of key articles focusing on *Aedes* mosquitoes in Africa and specifically on the surveillance of arboviral diseases, vector control and insecticide resistance. Reference lists of these articles were screened for potentially relevant citations missed by our electronic search. The references retrieved using the agreed search strategy were compiled, saved and de-duplicated in EndNote reference manager software, creating a review-specific database.

### Eligibility criteria

The scoping review included journal articles that were published from January 1980 to January 2023 on studies conducted on the geographical continent of Africa (including the French overseas departments of Mayotte and La Réunion) and published in any language. Included articles were relevant to one or more aspects of the review question, namely mosquito vector species being *Ae. aegypti*, *Ae. albopictus* or any of a list of 21 selected secondary *Aedes* vector species and topics of arboviruses, vector control, *Aedes* surveillance, insecticide resistance, vector bionomics, vector competency or distribution (with the last three topics reported in a second review article). Vector surveillance was defined as the routine, systematic monitoring of *Aedes* populations over a prolonged period (multiple years) to inform public health interventions. One-off cross-sectional or longitudinal studies to answer specific questions that were not described as being part of a wider surveillance system were not included in this review (but will be included under bionomics in the second review).

### Screening of eligibility

The EndNote database was exported to Rayyan systematic review software, allowing the review team to work together on the screening process. At the start of relevance screening, a sample of retrieved articles were screened by team members (BK, TAA, APY, JRB, JC, FA, BE, ANT, LAM, RT, MK) to ensure consistent application of the eligibility criteria and use of labels. Screening was conducted in pairs, first independently, then followed by a joint reconciliation of inclusion/exclusion decisions. In cases of disagreement, a third reviewer was enlisted to arbitrate the final decision. Relevance screening was performed on the title, abstract and keywords where available, to exclude articles that did not meet eligibility criteria. The full text of articles that met eligibility criteria were subject to a second round of screening by the review team using a pre-piloted, standardized spreadsheet data extraction tool developed prior to screening. Papers were excluded if they did not include primary data, were conference proceedings or abstracts, were not journal articles, the full text could not be obtained, mosquito species or disease were not of interest, subject was not of interest (not related to any of the six topics of interest in the two reviews), data were not collected in Africa or data were collected pre-1980.

## Results

**Fig. 1:**
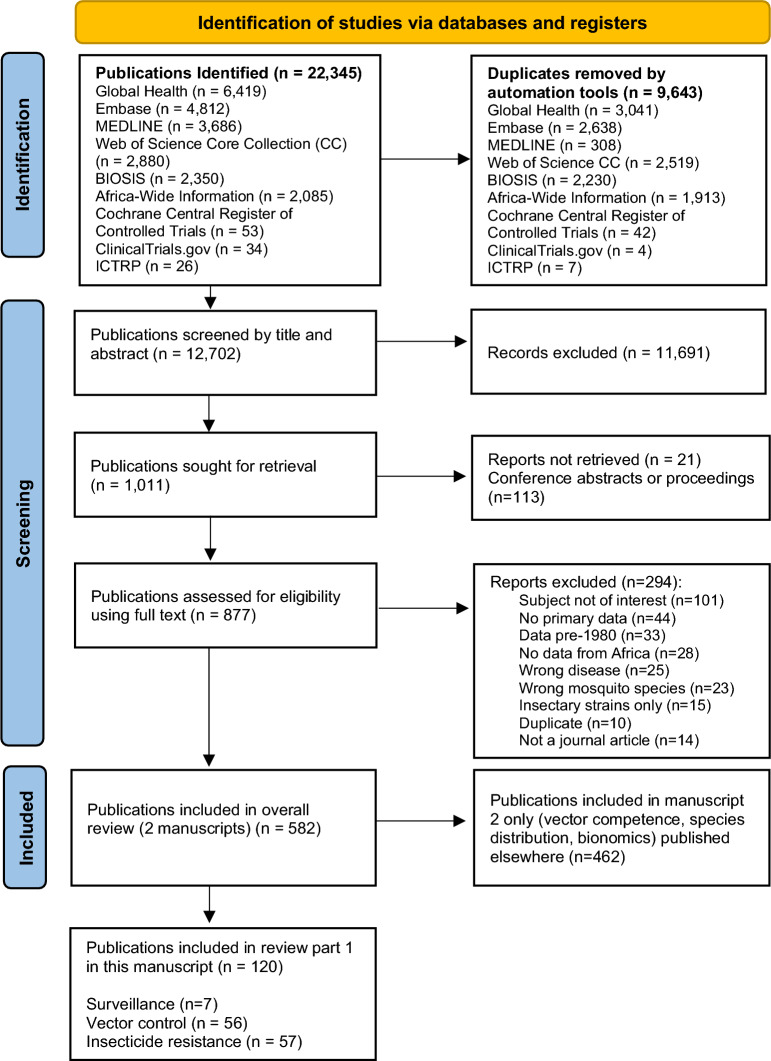
Preferred Reporting Items for Systematic Reviews and Meta-Analyses (PRISMA) flow chart illustrating the screening process for the scoping review, including sub-categorization of eligible publications containing surveillance, vector control or insecticide resistance data

Keyword searches of the databases yielded a total of 22,345 articles published between 1980 and 2023, from which 9643 duplicates were removed using automated EndNote tools described by Falconer [28], leaving 12,702 publications for screening by title and abstract (Fig. 1). Of those, 11,691 (92%) were excluded based on their title and abstract not meeting the inclusion criteria. Full texts were sought for 1011 publications of which 990 (98%) were successfully retrieved. Following full-text review, 582 were eligible for inclusion in the present review (533 were in English, 48 in French, 1 in Portuguese). Findings are reported in two parts: the first scoping review examined three topics: vector surveillance, vector control and insecticide resistance, and included 120 full text publications with information on one or more of these three topics. These 120 articles are summarized in Additional file 1: Table S1 (vector surveillance,* n* = 7); Additional file 2: Table S2 (vector control,* n* = 56); and Additional file 3: Table S3 (insecticide resistance,* n* = 57). Of those 120 articles, 46% (55/120) were published in the last five years covered by this review (2018–2023). The second scoping review on *Aedes* bionomics, vector competence and species distribution is reported separately elsewhere.

### Entomological surveillance of *Aedes* arbovirus vectors

Only seven publications from four of the 54 countries (7%) reviewed (Côte d’Ivoire,* n* = 1; Madagascar,* n* = 1; Senegal,* n* = 2; South Africa,* n* = 1) plus the territory of La Réunion (*n* = 2), featured data from routine *Aedes* surveillance systems. For the other 50 countries in Africa reviewed, none had no publications that met our definition of surveillance.

Two regions in Senegal had well-established mosquito arbovirus surveillance systems for early epidemic detection and the study of epizootic arbovirus cycles, both led by Institut Pasteur de Dakar [29]. The surveillance programme in Kedougou region (a hilly, forested area in south-east Senegal near the border with Guinea) has been operational since 1972, while surveillance in the Ferlo region (a semi-arid, flat area of Senegal) was established following the first outbreak of Rift Valley fever in 1988 [29, 30]. Traore-Lamizana et al*.* presented 1993 surveillance data from Kedougou region where human landing catches (HLCs) were conducted in forested areas (ground level and from 6- and 10-m-high platforms) and surrounding villages [30]. The primary purpose was to monitor sylvatic cycles of yellow fever to forecast epidemics as an indicator for West Africa. Yellow fever virus was detected in 187 pools of sylvatic *Aedes* from all four villages monitored (*Ae. furcifer*, *Ae. taylori* and *Ae. luteocephalus*) from all four villages monitored.. Ndiaye et al*.* reported data collected during 2012–2013, in which 10 arbovirus species (including West Nile, Rift Valley, Sindbis and several lesser known viruses) were detected in *Aedes* adults collected near ponds and villages surrounding Barkedji village (Ferlo region) [29]. Surveillance in the Ferlo region consisted of monthly HLCs, animal-baited light traps, and CO_2_-baited Centers for Disease Control and Prevention light traps (CDC-LT) at ground level.

In La Réunion, large-scale routine *Aedes* surveillance was established following major dengue (2004) and chikungunya (2005–2006) epidemics [31]. Two publications described *Aedes* (also reported as *Stegomyia*) indices derived from routine house inspection data by local representatives of the French Ministry of Health [31, 32]. Over 5 years of routine surveillance, 850,000 properties were inspected, with 70,000 positive for *Ae. albopictus* larvae or pupae [31]. Spatiotemporal monitoring of *Ae. albopictus* densities and breeding sites was conducted in La Réunion to determine the impact of control measures and health education campaigns [32].

In Madagascar, repeated *Aedes* surveillance was conducted from 1977 to 1986 on the island of Nosy-Be by the Institut Pasteur [33]. Surveillance consisting of CDC-LT trapping, HLCs and lemur (*Eulemur macaco*) landing catches resulted in the collection of 12 species of *Aedes* (with *Ae. albopictus* and *Ae. moucheti* being most common) but failed to isolate arboviruses from screened pools of mosquitoes, despite evidence from human and animal sera of arboviruses circulating in the area, including chikungunya, yellow fever, West Nile, dengue and Zika [33]. The initial establishment of focal surveillance systems was described in Côte d’Ivoire and South Africa. An arbovirus surveillance station was established through the Institut Pasteur in 1979 in Sokala-Sobara, Côte d’Ivoire, which collected dengue virus infection data in several sylvatic *Aedes* species [34]. However, this station was short-lived and is no longer operational (J Chabi, personal communication, 11 April 2025). In South Africa, the Durban Port Health Office conducted surveillance for invasive *Ae. albopictus* immatures in tyre shipments from Asia in the early 1990 s [35].

In conclusion, longitudinal arbovirus surveillance is greatly needed across a wider geographic area in Africa. In the absence of surveillance, periodic research studies can be valuable for characterizing local vector populations but cannot predict when outbreaks will occur. The sustained surveillance systems implemented in Senegal have been important for early epidemic detection and could be used as a model elsewhere, offering potential for knowledge sharing and regional capacity development.

### Control strategies for *Aedes* arbovirus vectors

**Fig. 2:**
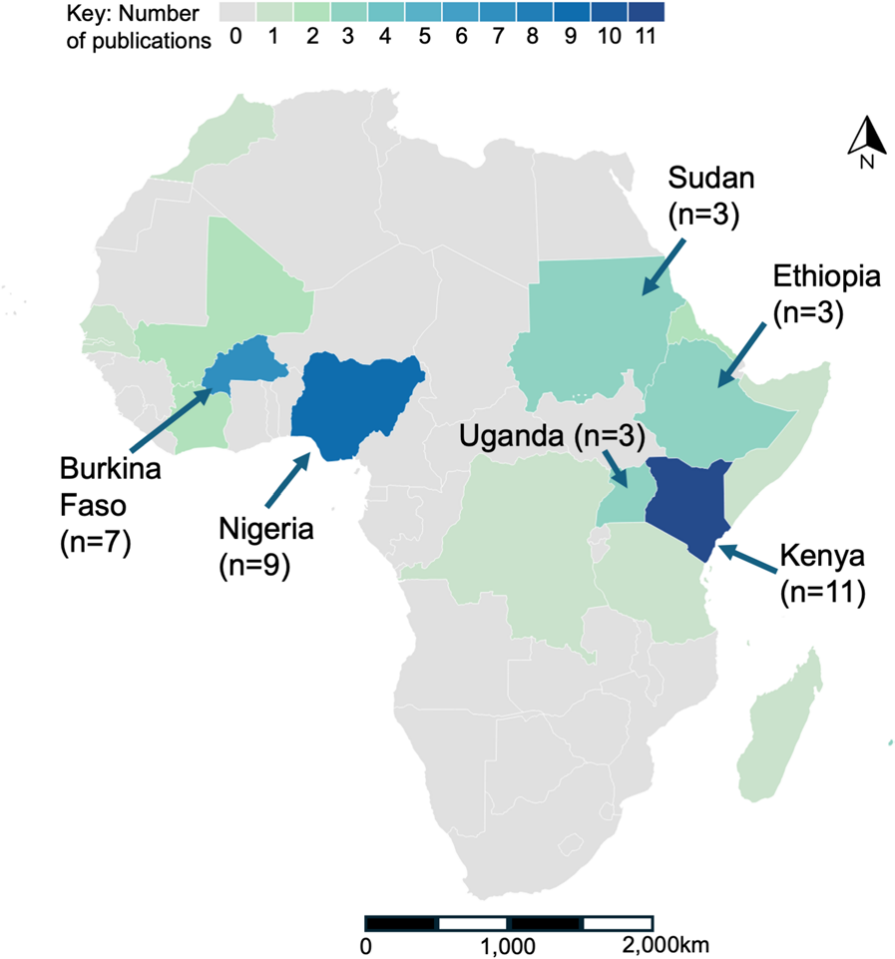
Number and geographical distribution of publications on *Aedes* vector control between 1980 and 2023, by country. Total publications = 56 (small islands not shown: Comoros*, n* = 1; Mauritius,* n* = 3; La Réunion,* n* = 3

There were 56 publications concerning *Aedes* vector control from 18 countries, with 10 countries, including the territory of La Réunion, having more than one publication (Fig. 2). Countries with the greatest number of publications were Kenya (*n* = 11), Nigeria (*n* = 9) and Burkina Faso (n = 7). In contrast, 36 of the 54 countries reviewed (67%) had no publications. This highlights the relative paucity of published *Aedes* vector control studies across most of Africa.

**Fig. 3:**
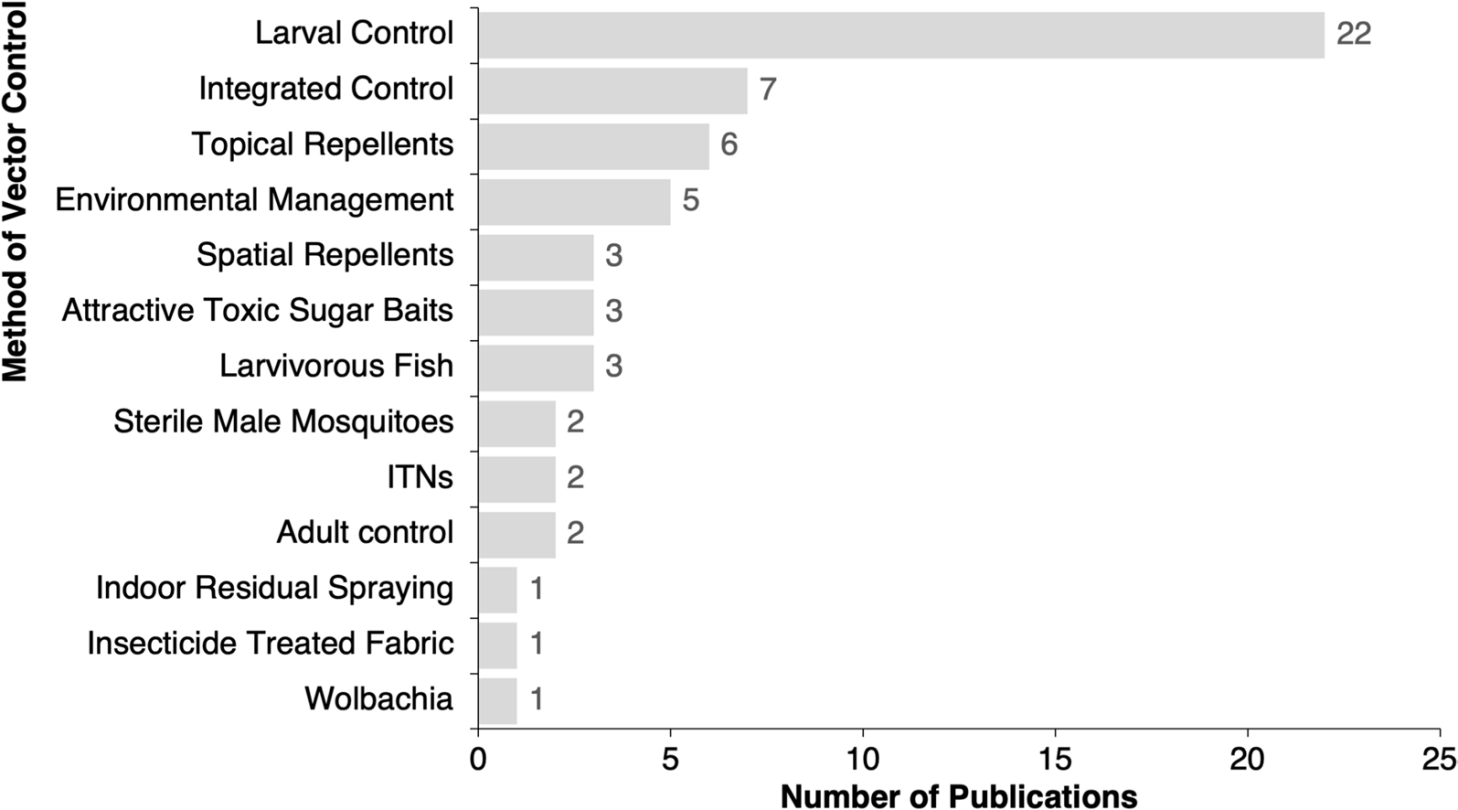
Number of publications reporting each *Aedes* vector control method. ITNs Insecticide-treated nets

The primary methods of *Aedes* vector control studied were larval control (*n* = 21), integrated control (defined as a combination of several control measures) (*n* = 7), topical repellents (*n* = 6), environmental management (*n* = 5) and spatial repellents (*n* = 3) (Fig. 3). A more detailed summary of vector control methods studied and type of study design used are shown in Table 1. Many of the publications reported the results of bioassays conducted under controlled conditions, or before and after field studies, compared to an untreated control at a limited number of sites. Entomological data were often limited in scale and/or scope, with no information on either human arboviral disease or arbovirus infection rates in mosquito populations. Only two vector control publications featured epidemiological endpoints (observational health facility-reported arbovirus cases) [37, 38]. Several studies involved well-known larvicides such as *Bacillus thuringiensis israelensis* (*Bti*), temephos, spinosad and methoprene, which all produced high levels of larval mortality or adult emergence inhibition in controlled bioassay studies [39–41]. Three studies in Uganda demonstrated the pathogenicity of *Coelomomyces indicus*, *Coelomomyces stegomyiae* and *Pythium* spp. fungal spores for larval control of *Ae. aegypti* in bioassays [42–44]. A number of studies investigated the potential of locally produced plant extracts (*n* = 5) and plant essential oils (*n* = 3) for larval control. The results indicated that several plant extracts and essential oils had larvicidal properties, including those from *Persea americana* (ground avocado seeds) [45], *Spondias mombin* (hog plum) [46] and *Cussonia barteri* [47]. Larvivorous fish reduced larval indices in two small-scale pilot studies in larger water storage containers (barrels, cisterns, wells) in Eritrea and Comoros but required frequent restocking [48, 49].

**Table 1:** Summary of publications that included vector control against *Aedes aegypti*

*Aedes* vector control strategy	Number of publications	Control method tested (no. of publications)	Type of study design (no. of publications)	Epidemiological outcomes reported
Larval control	21	*Bti* (7), plant extracts (5), fungal spores (4), essential oils (3), temephos (1), spinosad (1), methoprene (1)	Controlled bioassays^a^ (16), controlled before and after field study^b^ (3), controlled entomology field study^c^ (1), entomological RCT^d^ (1)	None
Integrated control	7	Combination of strategies (larvicides, ULV spraying, community education, source reduction)	Descriptive (3), uncontrolled entomology field study (2), controlled before and after study (1), entomological RCT (1)	Observational health facility arbovirus case data in Gould et al. [37] and Seidahmed et al. [38]
Topical repellents	6	DEET (2), picaridin (1), chromone derivatives (1), permethrin (1), plant extract (1)	Controlled bioassays (3), questionnaire (2), controlled entomological field study (1)	None
Environmental management	5	Community mobilization for source reduction (5)	Controlled before and after pilot study^e^(2), entomological RCT(1), questionnaire (1), baseline study (1)	None
Spatial repellents	3	Ethnomedicinal plant smoke (2), esbiothrin coils (1)	Controlled entomological field study (2), controlled bioassays (1)	None
Attractive toxic sugar baits	3	Dinotefuran (1), microencapsulated garlic oil (1)	Controlled before and after field study (3)	None
Larvivorous fish	3	*Poecilia reticulata* (1), *Aphanius dispar* (1), *Oreochromis niloticus* and *Tilapia zilli* (1)	Controlled before and after field study (1), pilot study (1), controlled bioassays (1)	None

All vector control publications studied *Ae. aegypti*, except in La Réunion where *Aedes albopictus* was the primary vector

*bti* *Bacillus thuringiensis israelensis*, ULV Ultra-low volume

^a^Controlled bioassays: experimental tests were done with wild collected mosquitoes under controlled conditions involving methods such as larval bioassay in containers or cone bioassay with incorporated untreated negative controls to allow for comparison

^b^Controlled before and after field study: entomological studies conducted before and after intervention in treated and untreated arms

^c^Controlled entomological field study: field study involving entomological methods such as human-biting rate, comparing treatment with untreated control

^d^RCT (randomized controlled trial): a study where participants or clusters are randomly assigned to intervention or control groups, with the aim to rigorously evaluate the effectiveness of vector control measures

^e^Pilot study: a small-scale rollout of the intervention in a field setting, generally intended to assess feasibility and guide larger-scale implementation

Given the tendency of *Ae. aegypti* and *Ae. albopictus* to breed in peridomestic containers, larval source reduction through environmental management featured in five publications as a stand-alone activity, and in a further seven as a component of integrated control (Table 1). Only a few of the methods for *Aedes* control have been evaluated using a rigorous design, such as randomized controlled trials (RCTs). A RCT in Kenya showed that improved caregiver knowledge regarding mosquito biology and control, coupled with source reduction, did not significantly reduce the number of household containers (potential larval habitats). This result occurred despite children and parents collecting 17,200 containers (1 ton of plastics), which were used to plant 4000 native trees as part of a community event [22]. A similar community-based intervention RCT study in Ouagadougou, Burkina Faso led to a modest reduction in residents’ exposure to vector saliva biomarkers and a decline in pupal index (pupae per 100 compounds examined) from 162 to 99 compared to 219 and 256 in the control neighbourhood [50, 51].

Spatial repellents release volatile chemicals into the air, creating a protective atmosphere in enclosed or semi-enclosed spaces. However, no large-scale studies or RCTs were identified in this review. Three studies were identified, all of which consisted of controlled bioassays or controlled entomology field studies involving the burning of fresh plants and plant powders or esbiothrin coils, with all showing some degree of short-term protection. Topical repellents applied directly to the skin featured in six studies, with DEET and picaridin reducing human-landing of *Ae. aegypti* in bioassay and small-scale field studies [52]. Completed questionnaires suggested that the use of skin repellent on La Réunion was not associated with reduced cases of chikungunya [53].

Four publications provided key insights into typical vector control responses implemented during arboviral epidemics in Africa (Table 2). In 2005, an outbreak of concurrent dengue, yellow fever and chikungunya was reported in South Kordofan State, Sudan, which resulted in 605 reported cases and thousands of suspected cases in the community [37]. A multipronged epidemic response was conducted by the Sudanese Ministry of Health with several international partners, which employed multiple vector control interventions, targeting both adults and larval stages, namely outdoor ground and aerial ultra-low volume (ULV) spraying and indoor ULV using pyrethroids; larviciding using temephos; and community education on larval source reduction related to water storage [37]. Similar integrated vector control was conducted in response to outbreaks of dengue and chikungunya in La Réunion [32], dengue in Port Sudan City, Sudan [38] and chikungunya in Dire Dawa, Ethiopia [54]. All vector control responses to arboviral disease outbreaks involved a combination of larviciding, ULV spraying (either indoors, outdoors or both) and community engagement to reduce larval sites, coupled with active source reduction by government workers (Table 2). In addition to the other interventions, ITNs were distributed to arbovirus patients in Port Sudan [38], and individual protection using topical repellents was strongly encouraged in La Réunion [32] and Sudan [38]. Nonetheless, despite the deployment of integrated vector management strategies, the impact of mosquito control on the outbreaks was largely inconclusive [37]. Control operations in Sudan were hampered by a lack of resources and personnel trained in mosquito control and entomological evaluation of control efficacy [37]. Vector control operations began after the transmission peak, when cases were already declining, and this trend may be attributable to the onset of the dry season, reduction in mosquito larval sites and population immunity rather than vector control or vaccination efforts [37]. In Dire Dawa, Ethiopia and Port Sudan, Sudan, before and after monitoring indicated a reduction in entomological indices (house, container, Breteau and pupae indices) following emergency vector control, but these assessments were too limited in design and scope to determine causality (lack of untreated control sites for comparison; short duration of monitoring [1 week after vector control in Ethiopia, 14 weeks in Sudan]; methods lacking detail so studies are not easily repeatable) [38, 54]. The need for better preparedness planning for arbovirus epidemics was described in all four publications, with La Réunion subsequently developing an arbovirus prevention plan following major dengue and chikungunya epidemics [32, 37, 38, 54].

**Table 2:** Summary of publications that describe emergency vector control measures in response to arbovirus epidemics

Location (reference)	Year of outbreak; virus type	Adult Mosquito Control	Larval control	Vector control scale and frequency	Environmental modification	Other	Entomological monitoring
Sudan: South Kordofan State [37]	2005 dengue; chikungunya, yellow fever	Ground and aerial space spraying, indoor ULV with pyrethroids (type not described)	Temephos (organo-phosphate) used for larval control	Two months of vector control Abu Jubaiyah and Rashad districts (towns and some villages)	Community education to encourage residents to manage water storage containers	NA	Ten towns surveyed during a two-week period. Larval and pupal collections, indoor PSC, aspirator, CDC-LT
Challenges: investigation began more than 1 month after the start of the dry season, and it was not possible to determine which mosquito vectors were involved in this outbreak; mosquito control limited primarily to larger towns despite most cases reported from the smaller villages; control operations hampered by a lack of resources and personnel trained in mosquito control and entomologic assessments of control efficacy; prompt recognition and control of outbreak hampered by lengthy delays in recognition of the outbreak and in shipping and testing of diagnostic samples							
La Réunion: island-wide [32]	2005–2007; dengue, chikungunya	Outdoor ULV with fenitrothion (organo-phosphate), deltamethrin or esbiothrin (pyrethroids)	Larviciding with temephos (2004–2006) or *Bti* (2006) using hand-held sprayers	Perifocal vector control, 10 houses around reported cases (2004 to January 2006), widespread control during peak of epidemic (February to May 2006)	Social mobilization aimed at cleaning the environment, protection from mosquito bites and reporting dengue-like symptoms	Topical repellents encouraged	House index, container index, Breteau index in 16 neighbourhoods initially, followed by 42 neighbourhoods island-wide. Monthly larval surveys in 60 household gardens per neighbourhood per month
Challenges: entomology surveillance had to be temporarily suspended due to the general increase in the refusal rate (from 17% in December 2004 to 26% in April 2005); the chikungunya epidemic revealed the need to develop an arbovirus prevention plan to enable faster, tailored intervention and to develop in-depth research on vectors, improve environmental and waste management and maintain regional health monitoring to best prevent epidemics							
Sudan: Port Sudan City [38]	2010; dengue type 3	Truck delivered ULV space spraying, indoor and outdoor thermal fogging with permethrin (pyrethroid)	Temephos applied to outdoor containers (mostly water storage drums);	Interventions implemented in foci where clusters of cases reported, vector control conducted for 14 weeks	Health worker-led source reduction in a diameter of 500 m from case clusters, house inspection & health education across the city to clean and cover pots and barrels	ITN distribution to patients, use of repellents encouraged	House index, container index, Breteau index, pupal/person index. 240 houses sampled per week (30 houses each from 8 sentinel sites) for aquatic stages. Monitoring began 2 months after the outbreak start
Challenges: deficiencies in the case reporting system; vector control stopped after 14 weeks due to budget constraints; lack of compliance of household members to regularly clean water storage containers; many windows/doors locked during ULV spraying; fogging machines regularly out of order; lack of susceptibility or efficacy testing of permethrin to local *Aedes* before operations							
Ethiopia: Dire Dawa City [54]	2019; chikungunya	Indoor and outdoor ULV with propoxur (carbamate)	Temephos used to treat stored water in containers that were difficult to remove	Not described	Community education how to manage breeding sites	NA	House index, Breateau index, container index, pupae index. 800 houses sampled (100 houses each in 8 kebeles), before and 1 week after vector control; resting collection of adult mosquitoes and collection of immature stages in/around households
Challenges: no national control system designed for *Aedes* control; irregular supply of piped drinking water, therefore the community stored rainwater for long periods of time for domestic use. Detection and early warning systems need to be strengthened. Chemicals and spray materials should be ready for prevention of mosquito density and mosquito borne diseases that will help in the immediate response to outbreak							

*Bti Bacillus thuringiensis israelensis*, *CDC-LT* Centers for Disease Control and Prevention light trap, *ITN* insecticide-treated net, NA not available, *PSC* pyrethrum spray catch, *ULV* ultra-low volume

### Insecticide resistance status of *Aedes* arbovirus vector species

**Fig. 4:**
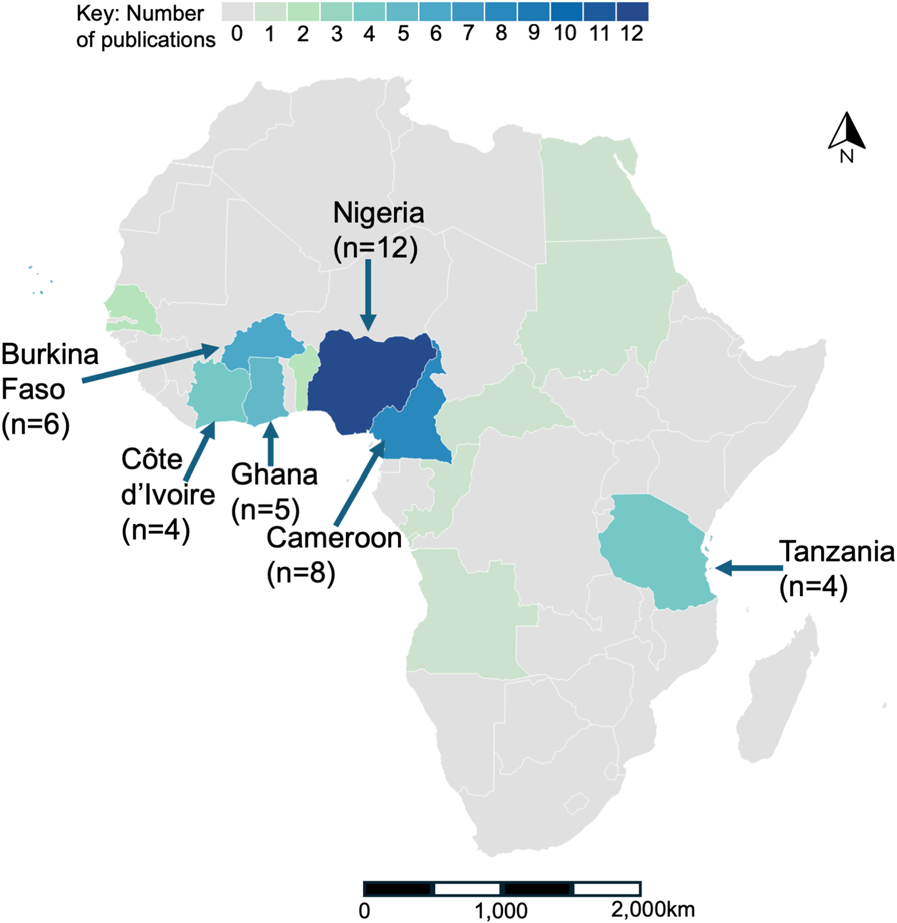
Number and geographical distribution of publications on *Aedes* insecticide resistance publications. Total publications = 57 (small island nations not shown: Cabo Verde, *n* = 4; La Réunion, *n* = 4)

*Aedes* insecticide resistance data was featured in 57 publications from 14 of the 56 (25%) countries in Africa reviewed, as shown in shown in Fig. 4. Countries with the greatest number of *Aedes* resistance publications were Nigeria (*n* = 12), Cameroon (*n* = 8), Burkina Faso (*n* = 6), Ghana (*n* = 5), Cabo Verde (*n* = 4), Tanzania (*n* = 4), Côte d’Ivoire (*n* = 4) and La Réunion (*n* = 4).

**Fig. 5:**
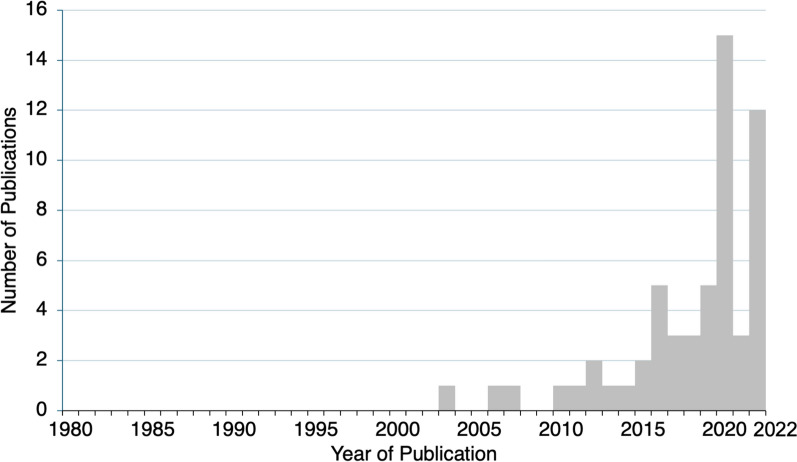
Number of *Aedes* insecticide resistance publications in Africa by year (1980–2022)

The number of publications has increased in recent years from no publications from 1980 through 2002, to 35 (61% of total publications) being published in the 4 years from 2019 through to 2022 (Fig. 5). The recent increase in studies related to *Aedes* insecticide resistance in Africa appears to be linked to an increase in arbovirus outbreaks. *Aedes* insecticide resistance studies from Cameroon [55], Burkina Faso [56], Nigeria [57], Ghana [58], La Réunion [59], Côte d’Ivoire [60], Tanzania [61], Senegal [62], Cabo Verde [63] and Mayotte [64] all reported recent outbreaks of *Aedes*-transmitted dengue, chikungunya, yellow fever or Zika viruses. A commonly cited reason for conducting *Aedes* insecticide resistance monitoring was to aid vector control decision-making and prepare for future arbovirus outbreaks, as emergency control measures depend heavily on the use of insecticides [58, 65]. Published studies were primarily conducted by research institutions, often by African institutions highly regarded for their expertise in malaria vector research.

### Adult insecticide resistance bioassays

**Fig. 6:**
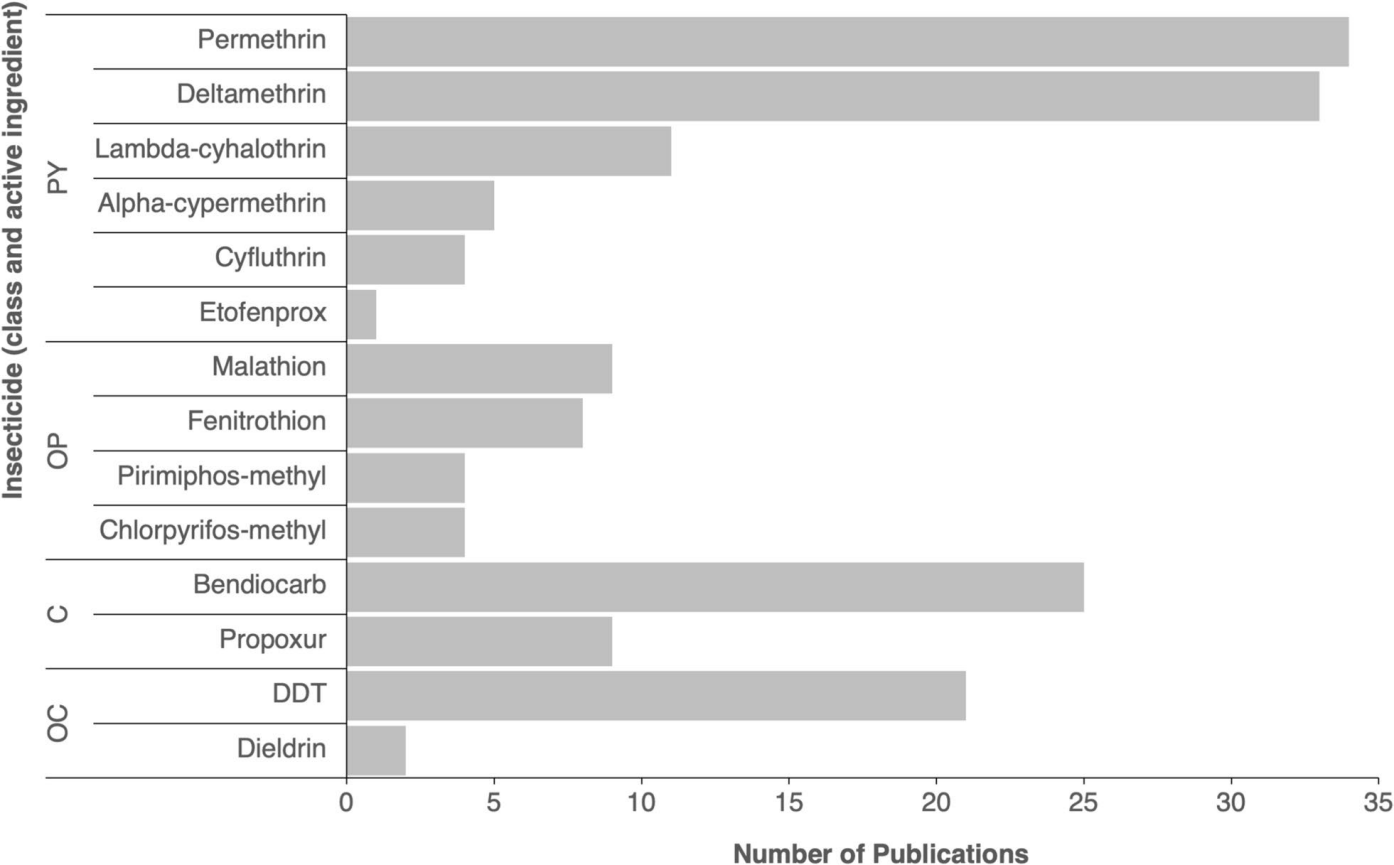
Insecticides tested to determine adult *Aedes aegypti* and *Aedes albopictus* insecticide susceptibility. C, Carbamate; OC, organochlorine; OP, organophosphate; *PY *pyrethroid

Bioassays primarily focused on adult mosquitoes (45/57 publications), using the WHO tube test as the method of choice (43/45), followed by bottle bioassays (2/45). *Aedes aegypti* (53 publications) and *Ae. albopictus* (12 publications) were the only species tested. *Aedes albopictus* susceptibility testing was conducted in Cameroon [55, 66–69], La Réunion [59, 70, 71], Republic of Congo [59] and Central African Republic [72]. A total of 14 insecticides were tested against adult *Ae. aegypti* and *Ae. albopictus* across all studies, including six pyrethroid, four organophosphate, two carbamate and two organochlorine insecticides. The most studied insecticides were permethrin and deltamethrin (pyrethroids), bendiocarb (carbamate) and dichlorodiphenyltrichloroethane (DDT, organochlorine) (Fig. 6). Pyrethroids were included in nearly all studies, but there was a relative lack of data on organophosphate resistance (only 9/57 studies tested malathion).

Based on a comprehensive multi-centre study, the WHO published updated guidance in 2022 regarding insecticide discriminating concentrations (defined as twice the lethal concentration 99 [LC_99_; dose required to kill 99% of target susceptible strain]) for *Ae. aegypti* and *Ae. albopictus* resistance testing [73]. Table 3 presents the doses of key pyrethroid and organophosphate insecticides, respectively, used in the studies identified in this review, with WHO discriminating concentrations underlined. Prior to the updated WHO guidance published in 2022, most studies were conducted using discriminating concentrations recommended for *Anopheles* mosquitoes (0.75% permethrin, 0.05% deltamethrin, 0.05% lambda-cyhalothrin and 5.0% malathion). Of the *Ae. aegypti* adult bioassays of permethrin, deltamethrin, lambda-cyhalothrin and malathion, 75% were overdosed compared to the new WHO guidance, with 11% underdosed (Table 3). The trend was similar for *Ae. albopictus* bioassays, albeit with fewer data points (18 vs 100 for *Ae. aegypti*).

**Table 3:** Insecticide doses used to determine adult *Aedes aegypti* and *Aedes albopictus* insecticide susceptibility

Insecticide (class)	Discriminating concentrations tested in publications (no. of studies)	
	*Aedes aegypti*	*Aedes albopictus*
Permethrin (pyrethroid)	0.025% (1) 0.25% (5) **0.40% (0)** 0.75% (31)	0.025% (1) 0.25% (2) **0.40% (0)** 0.75% (4)
Deltamethrin (pyrethroid)	**0.03% (5)** 0.05% (33) 0.06% (1)	**0.03% (2)** 0.05% (7) 0.06% (1)
Lambda-cyhalothrin (pyrethroid)	0.03% (2) **0.05% (9)** 0.08% (0) 0.5% (1)	0.03% (0) 0.05% (0) **0.08% (0)** 0.5% (0)
Malathion (organophosphate)	0.8% (3) **1.5% (0)** 5.0% (9)	0.8% (0) 1.5% (0) **5.0% (1)**

WHO discriminating concentrations are given in bold [73]

Most populations of *Ae. aegypti* were susceptible to malathion in WHO tubes when using a concentration of 5.0%. Given the updated WHO discriminating concentration is 1.5% for *Ae. aegypti*, resistance to this organophosphate has likely been underestimated [73]. In Senegal, resistance testing of *Ae. aegypti* was conducted using WHO tubes at nine locations, with a mean mortality of 99.8% with 5.0% malathion (indicating susceptibility) compared with a mean of 48.2% mortality with 0.8% malathion (indicating widespread resistance); these results highlight the importance of appropriate dosing during testing [62]. Adult bioassays of *Ae. aegypti* detected resistance to permethrin or deltamethrin in most of the countries tested, including Angola [74], Benin [75, 76], Burkina Faso [56, 77–80], Cameroon [55, 67, 81, 82], Côte d’Ivoire [60, 83], Egypt [84], Ghana [58, 85, 86], Republic of Congo [87], Nigeria [88], Senegal [62] and Tanzania [61, 89]. However, in countries where surveys were carried out in multiple locations countrywide (Cameroon, Côte d’Ivoire, Ghana, Republic of the Congo and Senegal), the results were variable, with some populations shown to be susceptible and others to be resistant. As the doses used were generally higher than the WHO discriminating concentrations, this is likely to be an underestimate of pyrethroid resistance.

### Insecticide resistance mechanisms

Molecular testing to determine mechanisms of insecticide resistance (mostly pyrethroid resistance) was conducted in 25 of the 56 (44%) publications, including some studies where no susceptibility testing was conducted. The resistance mechanisms detected according to country and *Aedes* species are summarized in Table 4. Several mutations in the voltage-gated sodium channel (VGSC) were detected in *Ae. aegypti* and *Ae. albopictus*. The most frequently reported VGSC mutation was F1534C (10 publications), which was present at high frequency in urban areas of Angola [74], Burkina Faso [56, 77], Cameroon [82] and Ghana [85]. The V1016I mutation was found at lower frequencies but often co-occurred with the F1534C mutation [55, 67, 78]. The V410L mutation was detected for the first time at high frequency in Luanda, Angola [74] and was subsequently reported in Cameroon [55, 67] and Burkina Faso [56]. Most publications did not conduct an analysis to determine the association of point mutations with phenotypic insecticide resistance, but there were a number of exceptions: In Ouagadougou, Burkina Faso, a significant association was found for the dual locus F1534C and V1016I genotypes with permethrin resistance [78]; in Luanda, there was a significant association between resistance phenotypes and genotypic frequencies for mutations V1016I and V410L, but not for F1534C [74]. Synergist bioassays conducted with piperonyl butoxide (PBO) demonstrated that mixed-function oxidases are important mechanisms for pyrethroid resistance in *Ae. aegypti* in Cameroon, Nigeria, Republic of the Congo, Côte d’Ivoire, Ghana and Burkina Faso, and for *Ae. albopictus* in Cameroon and Republic of the Congo (Table 4). Specific gene upregulation studies were less common and were only reported from Senegal and Cameroon, where several cytochrome P450 (CYP) enzymes were found to be significantly overexpressed (Table 4).

**Table 4:** Summary of resistance mechanisms reported in *Aedes aegypti* and *Aedes albopictus*

Resistance Mechanism	Number of publications	Countries where mechanism was present	Allele frequencies^a^/key findings	References
*Voltage-gated sodium channel mutations*				
F1534C	10	Angola, Burkina Faso, Cabo Verde, Cameroon, Ghana, Nigeria	Near fixation in *Ae. aegypti* populations in urban Ouagadougou (Burkina Faso) and Douala (Cameroon); high frequencies^a^ were reported in urban areas including Luanda (Angola), Dschang, Maroua (Cameroon), Banfora (Burkina Faso), Accra, Kumasi (Ghana); low frequencies^a^ reported in Kano (Nigeria), Cabo Verde and several locations in Cameroon	[55–57, 67, 74, 77, 78, 81, 82, 85]
V1016I	8	Angola, Burkina Faso, Cameroon, Ghana	High frequency in Luanda (Angola), Dschang (Cameroon); low frequencies in Ouagadougou, Banfora (Burkina Faso), several locations in Cameroon, and only one heterozygote reported from Accra (Ghana)	[55, 56, 67, 74, 77, 78, 82, 85]
V410L	4	Angola, Burkina Faso, Cameroon	Detected at high frequency in Luanda (Angola) and present at low frequency in Burkina Faso and Cameroon	[55, 56, 67, 74]
V1016G	2	Cameroon, Tanzania	Only detected in Douala (Cameroon) and Zanzibar (Tanzania)	[61, 82]
*Gene upregulation*				
MFOs (P450s)	2	Burkina Faso, Central African Republic	General non-specific upregulation of aggregate P450s	[72, 77]
PBO synergist bioassays^b`^	13	Burkina Faso, Cameroon, Côte d’Ivoire, Ghana, Nigeria, Republic of Congo	Significant increase in pyrethroid mortality following pre-exposure with PBO for *Ae. aegypti* in all 6 countries (in some, but not all sites) and for *Ae. albopictus* in Cameroon and Republic of the Congo	[55–58, 60, 67, 69, 77, 81, 87, 88, 127, 128]
CYP (several)	3	Cameroon, Senegal	CYP6BB2, CYP9J26, CYP9J32, CYP9J28, CCEAE3A significantly overexpressed throughout Senegal; CYP9J32, CYP9J28, CYP9M6 highly overexpressed in Douala and Yaoundé (Cameroon)	[62, 67, 82]
CYP9J10	1	Cameroon	CYP9J10 overexpressed in Douala (Cameroon)	[67]
CYP6P12	1	Cameroon	Overexpressed in *Ae. albopictus* in Douala and Yaoundé (Cameroon)	[67]
Alpha and beta esterases	2	Central African Republic, Ghana	Overexpression of alpha and beta esterases in *Ae. albopictus*, but only beta esterases in *Ae. aegypti* in Central African Republic (CAR). Decreased metabolic activity in Accra *Ae. aegypti* compared to susceptible strain	[58, 72]
GSTD4	2	Cameroon, Senegal	Significantly overexpressed throughout Senegal and in Douala (Cameroon)	[62, 82]
Reduced AChE sensitivity	1	Central African Republic	Detected in *Ae. aegypti* and *Ae. albopictus* in Central African Republic	[72]

*AChE* acetylcholinesterase, *CYP* cytochrome P450 enzyme, *GSTD4* Glutathione S-transferase Delta 4, *MFO* mixed function oxidases, *PBO* piperonyl butoxide

^a^Allele frequencies: near fixation > 90%, high frequencies > 50%, low frequencies < 50%

^b^PBO synergist bioassays are a proxy to detect metabolic upregulation of MFO

### Larval bioassays

**Fig. 7:**
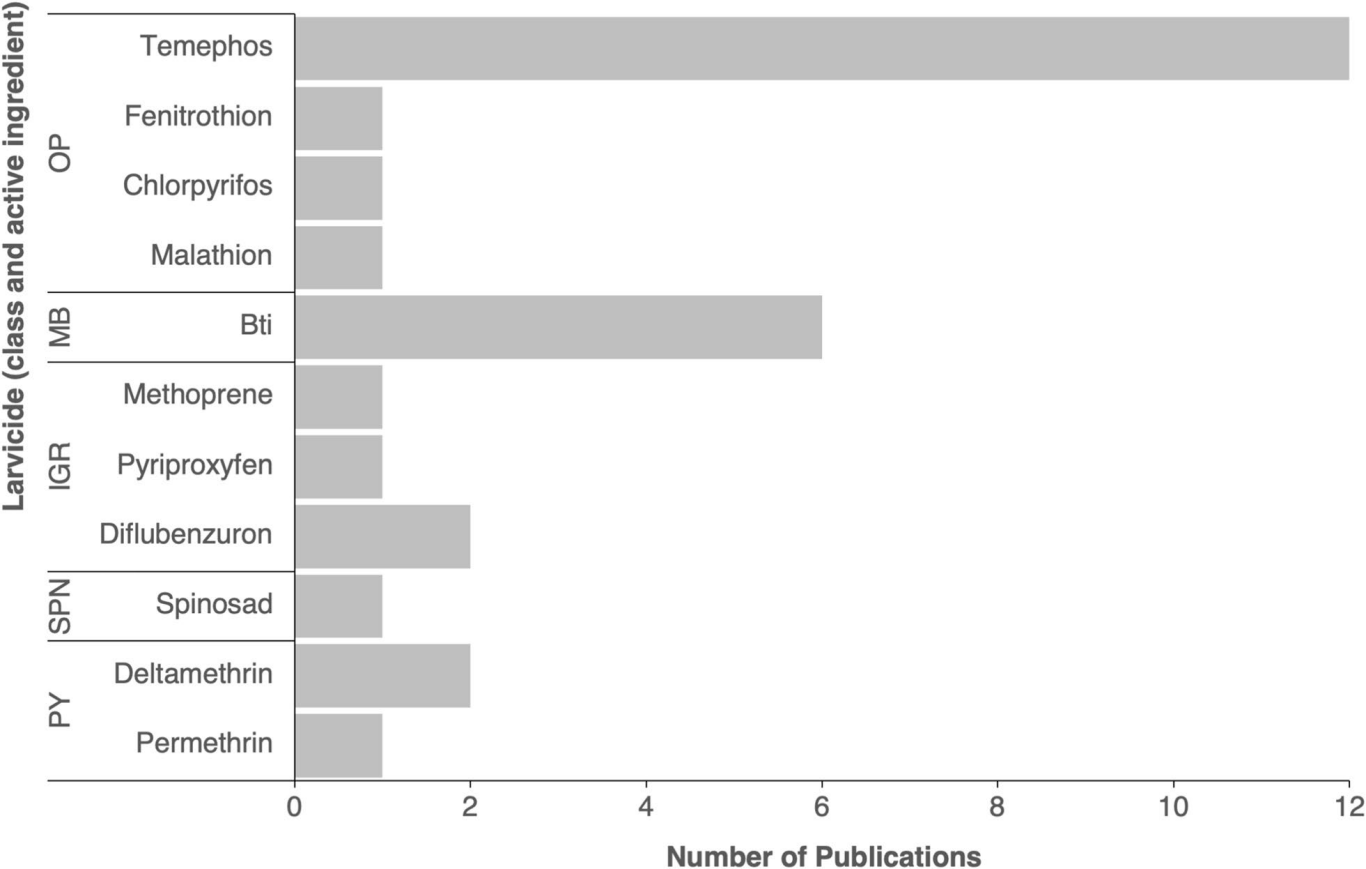
Larvicides tested to determine *Aedes aegypti* and *Aedes albopictus* susceptibility. IGR, Insect growth regulator; MB, microbial larvicide; OP, organophosphate; PY, pyrethroid; SPN, spinosyn

Larval insecticide resistance bioassays were uncommon (13/56 publications), despite larval control being a key recommended method for *Aedes* control and often utilized during emergency epidemic responses [37, 38]. Larvicides with WHO prequalification listing include formulations of organophosphates (temephos, pirimiphos-methyl), insect growth regulators (diflubenzuron, pyriproxyfen, novaluron), spinosyns (spinosad) and biological insecticides (*Bti*, *Lysinibacillus sphaericus* [*Ls*]) [90]. Figure 7 shows that temephos (*n* = 12) and *Bti* (*n* = 6) were the most frequently tested larvicides in susceptibility bioassays. Temephos has been used for *Aedes* control in several countries in Africa, including Sudan, Ethiopia, Cabo Verde and the departments of La Réunion and Mayotte [32, 37, 38, 54, 63, 64]. *Ae. aegypti* or *Ae. albopictus* were susceptible to temephos in Burkina Faso [77], Cameroon [66, 68, 81], Central African Republic [72], Republic of the Congo [87], Egypt [84], Nigeria [57] and Mayotte [64]. Cabo Verde was the only country where temephos resistance was reported, following several decades of use for vector control since 1979 [63]. Susceptibility to *Bti* was reported in Cabo Verde [63], Central African Republic [72] and Cameroon [66], with no resistant populations recorded in Africa. Populations of *Ae. aegypti* or *Ae. albopictus* demonstrated susceptibility to other less frequently tested larvicides (Fig. 7).

## Discussion

The WHO-coordinated Global Arbovirus Initiative and Global Strategic Preparedness, Readiness and Response Plan promotes preparedness of integrated vector control strategies [16, 91]. It is vital that countries prepare tailored arbovirus vector surveillance and control guidelines, based on locally collected data [92]. This review indicates that routine *Aedes* control outside of epidemic periods is uncommon in Africa, with exceptions being islands such as Cabo Verde and the French overseas departments of La Réunion and Mayotte. Sustained longitudinal *Aedes* vector surveillance was also uncommon, and only evident in Senegal and La Réunion, but this is likely an underestimate, as routine surveillance data are often unpublished and may only be accessible through government reports (which are often not publicly available). Initiatives such as the West African *Aedes* Surveillance Network (WAASuN), formed in 2017 to strengthen *Aedes* research, collaboration and capacity in 19 partner countries in West and Central Africa, demonstrate the growing recognition of improved arbovirus epidemic forecasting and preparedness to initiate prevention measures, especially vector control [93].

In most African countries, *Aedes* vector control is largely limited to reactive measures during major arbovirus epidemics. These responses typically involve multiple methods targeting both adult and larval stages, as described in Sudan, Ethiopia and La Réunion. However, compared to more established malaria control programmes there is a lack of organized, sustained control strategies and surveillance systems for arbovirus vectors. There is also a lack of evidence regarding the best practices and cost-effectiveness of integrated vector control in response to arbovirus epidemics in Africa, particularly when considering the reactive response and inevitable lag time between peak transmission and initiation of vector control. This deficiency reflects the challenges of undertaking research during the acute response phase of an outbreak, but also the lack of RCTs with epidemiological endpoints to determine which combination of interventions work best in non-epidemic periods [94]. A recent critical appraisal of the quality of studies measuring the effectiveness of vector control in Africa against *Ae. aegypti* and *Ae. albopictus* highlighted several common limitations, including lack of a control group, lack of blinding, short follow-up period post-intervention and type of outcome used for evaluation [95].

This limitation extends beyond Africa, with the authors of a recent review of *Ae. aegypti* vector control in continental USA concluding that there are limited evidence-based control recommendations or protocols in place [96]. There is also a need for more clarity on epidemic thresholds, which can be strengthened by local, data-driven determination, as described for dengue in Burkina Faso [97].

There are numerous outstanding operational questions surrounding vector control measures, both in epidemic and non-epidemic periods. For example, larval control is used globally for *Aedes* control, but there is a lack of a standardized approach [98]. Important parameters are needed to develop locally-tailored best practices for larviciding, including research on the longevity of vector suppression in specific settings to guide frequency of larvicide application; methods of larvicide application that optimize coverage; impact on *Aedes* indices under operational conditions on different *Aedes* species across urban, rural and sylvatic settings; and on arbovirus transmission during epidemic or longitudinal application. Since larval control is being employed in some urban settings in Africa against malaria vectors, there is potential for integrated vector management programmes to target both container and ground water breeding vectors [99–101]. Such efforts are even more urgent now, following the spread of the invasive urban malaria vector *Anopheles stephensi,* which commonly shares container habitats with *Ae. aegypti* and *Ae. albopictus,* through several African cities [102, 103]. In contrast, the impact of widespread use of ITNs for malaria vector control on indoor-resting *Aedes* remains unclear [11, 104].

Source reduction through environmental modification and waste management are appealing propositions as they can be undertaken with minimal training, do not require specialized equipment, offer co-benefits such as environmental clean-up and can be carried out by community mobilization or dedicated vector control personnel. It is important to promote the active involvement of municipalities in fulfilling their responsibilities to organize well-planned and budgeted environmental sanitation programs, including the provision of reliable water systems, waste disposal systems and plastic recycling [105]. Despite environmental modification being a proven method when thoroughly conducted [106], important questions remain about implementation approaches, particularly with the increasing abundance of discarded plastic containers in Africa. Strong community engagement through respected leaders is an essential component of effective public health strategies [107]. While community mobilization for source reduction is a strategy frequently advocated, there are several behavioural, cultural and biological challenges (e.g. cryptic larval sites) that require further investigation [108, 109].

This review identified several controlled bioassay studies of locally prepared plant extracts. However, before such biological products can be deployed on a broader scale, more comprehensive efficacy studies are needed to assess duration of action, human and environmental safety and strategies for quality-assured community-based or commercial production. New tools for *Aedes* control are being developed globally, including *Wolbachia*-infected mosquito releases for dengue control [110], spatial repellents [111], various genetically engineered *Ae. aegypti* [112, 113] and lethal oviposition traps [114]. However, none of these approaches had been fully evaluated in Africa during the period covered in this review. Since then, evaluation of *Wolbachia*-infected *Aedes aegypti*—an intervention shown to be effective in reducing dengue transmission in an RCT in Indonesia [115]—is underway in Burkina Faso [116].

With the increasing frequency and severity of arbovirus epidemics and growing urban populations in Africa, countries must prepare plans for epidemic emergency response. Insecticide resistance monitoring is a vital component and should always be used to guide optimal choice of insecticides for control programmes. In this review, the criteria for insecticide choice in adult *Aedes* resistance studies were often unclear and rarely linked with the planning of *Aedes* vector control operations. Organochlorines and carbamates were frequently included in publications, despite being unsuitable or unlikely options for *Aedes* adult control. DDT and dieldrin are persistent organic pollutants that should not be used for arbovirus epidemic response [117]. Targeted IRS, where primary *Ae. aegypti* resting locations (lower walls [< 1.5 m] and under beds and furniture) are sprayed, has shown potential in studies outside Africa [118, 119], but has yet to be tried in the African context. While IRS with more suitable insecticides, known as “IRS for urban *Aedes* control”, is recommended by WHO for integrated control of arboviruses, it is unlikely to be prioritized in Africa, with IRS programmes historically focused in rural areas with a high malaria burden [11, 120].

Globally, adult control of *Aedes* mosquitoes is often conducted using repeated aerial or ground-based ULV space sprays [121]. Insecticides commonly used for ULV application belong to two classes of chemistry: pyrethroids (PY) and organophosphates (OP). Space sprays with WHO prequalification listing include formulations of malathion (OP) and PYs: deltamethrin, lambda-cyhalothrin and prallethrin [122]. Therefore, we recommend that insecticide resistance studies on adult *Aedes* should prioritize OP and PY insecticides (with and without PBO synergists), particularly insecticides that have the highest likelihood of being used for emergency epidemic response. This strategy should be tailored by country depending on the products locally registered. There are more recent bi-formulated products which include imidacloprid (neonicotinoid) + prallethrin (PY) and flupyradifurone (butanolide) + transfluthrin (PY) [122]. An outdoor ULV formulation containing three active ingredients, namely abamectin (avermectin), fenpropathrin (PY) and C-8910 (fatty acid blend), is also currently under assessment for WHO prequalification [123]. These insecticides should be considered for expanded testing, especially if resistance to OPs and lack of PBO synergism to PYs is detected.

As the WHO discriminating concentrations for *Ae. aegypti* and *Ae. albopictus* are substantially lower than those for *Anopheles*, many studies conducted prior to 2021 are likely to have underestimated the level of insecticide resistance present in *Aedes* populations. While it is important that WHO have rigorously determined species-specific diagnostic doses, it may prove to be a cost and logistics barrier, as a dedicated budget and procurement plans will be required. Most countries in Africa conduct annual susceptibility testing of *Anopheles* using WHO insecticide papers to inform malaria vector control decision-making. The availability of insecticide-treated papers meant that testing of *Aedes* was possible without significant additional funding (several of the publications were unfunded or had low-level institutional funding).

Larval susceptibility testing was rarely conducted but should be done regularly in locations where active larval control is being undertaken or as baseline preparation for future control or epidemic response measures. A complicating factor of larvicide susceptibility tests is that standardized diagnostic doses have not been established by the WHO for most larvicides. The limited guidance for larvicide diagnostic doses is reflected in the wide range of doses used in publications, as has been described in other regions [124]. Temephos was the most tested larvicide, and has also been used focally for decades in Africa for control of copepods in drinking water sources, as part of the global Guinea Worm Eradication Program (GWEP) [125]. However, the emergence of temephos resistance in Cabo Verde is a reminder of the need for frequent monitoring [63]. *Bacillus thuringiensis israelensis* (*Bti*) is often the first-line larvicide for *Ae. aegypti* control globally due to its effectiveness and highly specific mechanism of action. A further advantage is that *Bti* consists of four distinct toxins (Cry4Aa, Cry4Ba, Cry11Aa and Cyt1Aa), meaning that resistance should develop much more slowly than to conventional insecticides, and has only rarely been reported globally [126]. Limitations include the partial duration of action and loss of efficacy in organically polluted water [98]. Therefore, more work is needed to tailor optimal larvicide choice by setting.

## Conclusions

This review summarizes the current state of knowledge on arbovirus vector surveillance, vector control and insecticide resistance monitoring in Africa. It serves as a foundation for engaging stakeholders and advancing the research agenda toward strengthening evidence-based practices and standards for detecting and controlling arbovirus epidemics.

**Table:** 

Topic	Key recommendations
Vector surveillance	• Longitudinal arbovirus surveillance is greatly needed across a wider geographic area in sub-Saharan Africa • Sustained surveillance systems implemented in Senegal are important for early epidemic detection and can be used as a model elsewhere, offering great potential for regional capacity development
Vector control	• With the increasing frequency and severity of arbovirus epidemics, countries should prepare locally tailored plans for arbovirus vector control, including epidemic response strategies • More high-quality evidence is needed regarding best practices and cost-effectiveness of vector control in response to arbovirus epidemics in Africa
Insecticide resistance	• Insecticide resistance studies of adult *Aedes* should prioritize organophosphate and pyrethroid insecticides (with/without PBO synergist), particularly insecticides that have the highest likelihood of being used for emergency epidemic response (ULV sprays) • Adult *Aedes* susceptibility tests should be conducted using the updated WHO discriminating concentrations published in 2022 • As larval control is often utilized during emergency epidemic responses, larval susceptibility tests should be conducted to guide vector control plans • Wider geographical sampling of *Aedes* populations is needed to better understand the selection of insecticide resistance mechanisms and how these varying profiles affect the efficacy of vector control

*PBO* Piperonyl butoxide,* ULV* ultra-low volume

## Supplementary Information

Additional file 1: Table S1.

Additional file 2: Table S2.

Additional file 3: Table S3.

## Data Availability

All data sets are included in this published article and its supplementary information files.

## Abbreviations

*Bti*: *Bacillus thuringiensis israelensis*
CDC-LT: Centers for Disease Control and Prevention light trap
CYP: Cytochrome P450 enzymes
DDT: Dichlorodiphenyltrichloroethane
GWEP: Guinea Worm Eradication Program
HLC: Human landing catch
IRS: Indoor residual spraying
LC: Lethal concentration
*Ls*: *Lysinibacillus sphaericus*
LSHTM: London School of Hygiene and Tropical Medicine
OP: Organophosphate
PBO: Piperonyl butoxide
PY: Pyrethroid
RCT: Randomized controlled trial
ULV: Ultra-low volume
WAASun: West African *Aedes* Surveillance Network
