# Supplementary material for: Building resilience against the growing threat of arboviruses: a scoping review of Aedes vector surveillance, control strategies and insecticide resistance in Africa
Source: Parasit Vectors. 2025 Oct 17;18:415. doi: 10.1186/s13071-025-07049-7 (PMC12535094; doi:10.1186/s13071-025-07049-7)
Supplement: Supplementary file 1 — Additional file 1: Table S1. [file 13071_2025_7049_MOESM1_ESM.docx]

| **Supplementary Table 1. Vector Surveillance Publications** | | | | |
| --- | --- | --- | --- | --- |
| Number | Study | Publication Year | Country | Ref |
| 1 | Ndiaye et al. | 2018 | Senegal | [1] |
| 2 | Boyer et al. | 2014 | La Réunion | [2] |
| 3 | Dehecq et al. | 2008 | La Réunion | [3] |
| 4 | Traore-Lamizana et al. | 1996 | Senegal | [4] |
| 5 | Jupp et al. | 1992 | South Africa | [5] |
| 6 | Fontenille et al. | 1988 | Madagascar | [6] |
| 7 | Cordellier et al. | 1983 | Côte d’Ivoire | [7] |

Table S1 shows the seven publications that met our inclusion criteria for vector surveillance. Vector surveillance was defined as the routine, systematic monitoring of *Aedes* populations over a prolonged period (multiple years) to inform public health interventions. One-off cross-sectional or longitudinal studies to answer specific questions that were not described as being part of a wider surveillance system were not included in this review (but will be included under bionomics in the second review).

**References**

1. Ndiaye EH, Diallo D, Fall G, Ba Y, Faye O, Dia I, Diallo M: **Arboviruses isolated from the Barkedji mosquito-based surveillance system, 2012-2013.** *BMC Infect Dis* 2018, **18:**642.

2. Boyer S, Foray C, Dehecq JS: **Spatial and temporal heterogeneities of Aedes albopictus density in La Reunion Island: rise and weakness of entomological indices.** *PLoS One* 2014, **9:**e91170.

3. Dehecq J-S, Fohr G, Thiria J: **Plan de lutte contre Aedes albopictus pendant l’épidémie de chikungunyaà La Réunion en 2005-2007.** Bulletin Epidemiologique Hebdomadaire2008.

4. Traoré-Lamizana M, Fontenille D, Zeller HG, Mondo M, Diallo M, Adam F, Eyraud M, Maiga A, Digoutte JP: **Surveillance for yellow fever virus in eastern Senegal during 1993.** *J Med Entomol* 1996, **33:**760-765.

5. Jupp PG, Kemp A: **Aedes albopictus and other mosquitoes imported in tires into Durban, South Africa.** *J Am Mosq Control Assoc* 1992, **8:**321-322.

6. Fontenille D, Mathiot C, Rodhain F, Coulanges P: **[Arbovirus infections on the island of Nosy-Be; serologic and entomologic findings].** *Arch Inst Pasteur Madagascar* 1988, **54:**101-115.

7. Cordellier R, Bouchité B, Roche J-C, Monteny N, Diaco B, Akoliba P: ***Circulation selvatique du virus Dengue 2  en 1980, dans les savanes sub-soudaniennes  de Côte d'Ivoire*.** *Cahier ORSTOM, sér Ent méd et Parasitol* 1983, **vol. XXI**.
