# Supplementary material for: Building resilience against the growing threat of arboviruses: a scoping review of Aedes vector surveillance, control strategies and insecticide resistance in Africa
Source: Parasit Vectors. 2025 Oct 17;18:415. doi: 10.1186/s13071-025-07049-7 (PMC12535094; doi:10.1186/s13071-025-07049-7)
Supplement: Supplementary file 2 — Additional file 2: Table S2. [file 13071_2025_7049_MOESM2_ESM.docx]

| **Supplementary Table 2. Vector Control Publications** | | | | | |
| --- | --- | --- | --- | --- | --- |
| **Number** | **Study** | **Publication Year** | **Country** | **Vector Control Strategy** | **Ref** |
| 1 | Derua et al. | 2022 | Tanzania | Larval control | [1] |
| 2 | Forsyth et al. | 2022 | Kenya | Environmental management | [2] |
| 3 | Mukhtar et al. | 2022 | Nigeria | ITNs | [3] |
| 4 | Wendimu et al. | 2021 | Ethiopia | Spatial repellents | [4] |
| 5 | Dambach et al. | 2021 | Burkina Faso | Larval control | [5] |
| 6 | Waldetensai et al. | 2021 | Ethiopia | Integrated control | [6] |
| 7 | Yameogo et al. | 2021 | Burkina Faso | Larval and adult control | [7] |
| 8 | Yenus et al. | 2021 | Eritrea | Integrated control | [8] |
| 9 | Onwuzulike et al. | 2021 | Nigeria | IRS | [9] |
| 10 | Ranarijaona et al | 2021 | Madagascar | Topical repellents | [10] |
| 11 | Bonnet et al. | 2020 | Burkina Faso | Environmental management | [11] |
| 12 | Forsyth et al. | 2020 | Kenya | Environmental management | [12] |
| 13 | Iyaloo et al. | 2019 | Mauritius | Sterile male mosquitoes | [13] |
| 14 | Sissoko et al. | 2019 | Mali | Attractive toxic sugar baits | [14] |
| 15 | Culbert et al. | 2018 | Sudan | Sterile male mosquitoes | [15] |
| 16 | Ouedraogo et al. | 2018 | Burkina Faso | Environmental management | [16] |
| 17 | Yalwala et al. | 2016 | Kenya | Attractive toxic sugar baits | [17] |
| 18 | Nzelibe et al. | 2015 | Nigeria | Larval control | [18] |
| 19 | Eze et al. | 2014 | Nigeria | Larval control | [19] |
| 20 | Goorah et al. (a) | 2014 | Mauritius | Environmental management | [20] |
| 21 | Goorah et al. (b) | 2014 | Mauritius | Topical repellents | [21] |
| 22 | Kemabonta et al. | 2013 | Nigeria | Larval control | [22] |
| 23 | Edwin et al. | 2013 | Nigeria | Larval control | [23] |
| 24 | Khallaayoune et al. | 2013 | Morocco | Attractive toxic sugar baits | [24] |
| 25 | Seidahmed et al. | 2012 | Sudan | Integrated control | [25] |
| 26 | Mousson et al. | 2012 | La Réunion | Wolbachia | [26] |
| 27 | Dube et al. | 2011 | Ethiopia | Spatial repellents | [27] |
| 28 | Tun-Lin et al. | 2009 | Kenya | Integrated control | [28] |
| 29 | Seye et al. | 2009 | Senegal | Larval and adult control | [29] |
| 30 | Staikowsky et al. | 2008 | La Réunion | Topical repellents | [30] |
| 31 | Gould et al. | 2008 | Sudan | Integrated control | [31] |
| 32 | Dehecq et al. | 2008 | La Réunion | Integrated control | [32] |
| 33 | Dondji et al. | 2005 | Burkina Faso | Larval control | [33] |
| 34 | Badolo et al. | 2004 | Burkina Faso | Topical repellents | [34] |
| 35 | Bassole et al. | 2003 | Burkina Faso | Larval control | [35] |
| 36 | Konan et al. | 2003 | Côte d’Ivoire | Topical repellents | [36] |
| 37 | Diallo et al. | 2001 | Mali | Larval control | [37] |
| 38 | Obeta et al. | 1998 | Nigeria | Larval control | [38] |
| 39 | Horosko et al. | 1996 | Somalia | Integrated control | [39] |
| 40 | Karch et al. | 1995 | DR Congo | ITNs | [40] |
| 41 | Asimeng et al. | 1993 | Kenya | Larvivorous fish | [41] |
| 42 | Fletcher et al. | 1992 | Eritrea | Larvivorous fish | [42] |
| 43 | Logan et al. | 1992 | Kenya | Larval control | [43] |
| 44 | Mutinga et al. | 1992 | Kenya | Insecticide-treated fabric | [44] |
| 45 | Mwangi et al. | 1992 | Kenya | Larval control | [45] |
| 46 | Ejiofor et al. | 1991 | Nigeria | Larval control | [46] |
| 47 | Logan et al. | 1990 | Kenya | Larval control | [47] |
| 48 | Ejiofor et al. | 1989 | Nigeria | Larval control | [48] |
| 49 | Lindsay et al. | 1989 | The Gambia | Topical repellents | [49] |
| 50 | Sabatinelli et al. | 1988 | Comoros | Larvivorous fish | [50] |
| 51 | Birley et al. | 1987 | Kenya | Spatial repellents | [51] |
| 52 | Nnakumusana et al. (a) | 1987 | Uganda | Larval control | [52] |
| 53 | Nnakumusana et al. (b) | 1987 | Uganda | Larval control | [53] |
| 54 | Jondiko et al. | 1986 | Kenya | Larval control | [54] |
| 55 | Hougard et al. | 1985 | Côte d’Ivoire | Larval control | [55] |
| 56 | Nnakumusana et al. | 1985 | Uganda | Larval control | [56] |

Table S2 shows the 56 publications that met our inclusion criteria for *Aedes* vector control. These studies were analyzed to determine the primary methods of *Aedes* vector control in Africa and evidence for their effectiveness; effects of malaria vector control interventions on *Aedes* vector populations, and observations that indicate the role of vector control in the prevention and control of arboviral outbreaks.

**References**

1. Derua YA, Tungu PK, Malima RC, Mwingira V, Kimambo AG, Batengana BM, Machafuko P, Sambu EZ, Mgaya YD, Kisinza WN: **Laboratory and semi-field evaluation of the efficacy of.** *Curr Res Parasitol Vector Borne Dis* 2022, **2:**100089.

2. Forsyth JE, Kempinsky A, Pitchik HO, Alberts CJ, Mutuku FM, Kibe L, Ardoin NM, LaBeaud AD: **Larval source reduction with a purpose: Designing and evaluating a household- and school-based intervention in coastal Kenya.** *PLoS Negl Trop Dis* 2022, **16:**e0010199.

3. Mukhtar MM, Ibrahim SS: **Temporal Evaluation of Insecticide Resistance in Populations of the Major Arboviral Vector.** *Insects* 2022, **13**.

4. Wendimu A, Tekalign W: **Field efficacy of ethnomedicinal plant smoke repellency against.** *Heliyon* 2021, **7:**e07373.

5. Dambach P, Bärnighausen T, Yadouleton A, Dambach M, Traoré I, Korir P, Ouedraogo S, Nikiema M, Sauerborn R, Becker N, Louis VR: **Is biological larviciding against malaria a starting point for integrated multi-disease control? Observations from a cluster randomized trial in rural Burkina Faso.** *PLoS One* 2021, **16:**e0253597.

6. Waldetensai A, Gemechu F, Kinfe E, Amare H, Hagos S, Teshome D, Hailemariam A, Nigatu W, Tadesse A, Keneni D, et al: **Aedes mosquito responses to control interventionsagainst the Chikungunya outbreak of Dire Dawa, Eastern Ethiopia.** *International Journal of Tropical Insect Science* 2021.

7. Yameogo F, Wangrawa D, Sombie A, Sanon A, Badolo A: **Insecticidal activity of essential oils from six aromatic plants against Aedes aegypti, dengue vector from two localities of Ouagadougou, Burkina Faso.** *Arthropod - Plant Interactions* 2021, **15**.

8. Yenus M, Berhe M, Brhane M, Kiflemariam A, Sereke K: **Assessment the effectiveness of community based dengue and chikungunya intervention: Keren, Anseba zone Eritrea (2020).** *International Journal of Mosquito Research* 2021, **8**.

9. Onwuzulike I, Onyebueke A, Irikannu K, Nzeukwu C, Ogbonna C, Nwangwu R, Ochiaka C: **Relative abundance and diversity of man-biting mosquito species before and after indoor residual spraying programme in Awka and Environs, Anambra State, Nigeria.** *Trends in Entomology* 2021, **17**.

10. Ranarijaona MM, Rambala Rakotomena NAH, Andrianjafy MT, Ramiharimanana FD, Herinirina LC, Ramarosandratana NH, Briou B, Fajardie P, Mavingui P, Métay E, et al: **Development of Sustainable Chemistry in Madagascar: Example of the Valuation of CNSL and the Use of Chromones as an Attractant for Mosquitoes.** *Molecules* 2021, **26**.

11. Bonnet E, Fournet F, Benmarhnia T, Ouedraogo S, Dabiré R, Ridde V: **Impact of a community-based intervention on Aedes aegypti and its spatial distribution in Ouagadougou, Burkina Faso.** *Infect Dis Poverty* 2020, **9:**61.

12. Forsyth JE, Mutuku FM, Kibe L, Mwashee L, Bongo J, Egemba C, Ardoin NM, LaBeaud AD: **Source reduction with a purpose: Mosquito ecology and community perspectives offer insights for improving household mosquito management in coastal Kenya.** *PLoS Negl Trop Dis* 2020, **14:**e0008239.

13. Iyaloo D, Damiens D, Facknath S, Elahee K, Bheecarry A: **Dispersal and survival of radio-sterilised male Aedes albopictus Skuse (Diptera: Culicidae) and estimation of the wild populations in view of an sterile insect technique programme in Pointe des Lascars, Mauritius.** *International Journal of Tropical Insect Science* 2019, **39**.

14. Sissoko F, Junnila A, Traore MM, Traore SF, Doumbia S, Dembele SM, Schlein Y, Traore AS, Gergely P, Xue RD, et al: **Frequent sugar feeding behavior by Aedes aegypti in Bamako, Mali makes them ideal candidates for control with attractive toxic sugar baits (ATSB).** *PLoS One* 2019, **14:**e0214170.

15. Culbert NJ, Maiga H, Somda NSB, Gilles JRL, Bouyer J, Mamai W: **Longevity of mass-reared, irradiated and packed male Anopheles arabiensis and Aedes aegypti under simulated environmental field conditions.** *Parasit Vectors* 2018, **11:**603.

16. Ouédraogo S, Benmarhnia T, Bonnet E, Somé PA, Barro AS, Kafando Y, Soma DD, Dabiré RK, Saré D, Fournet F, Ridde V: **Evaluation of Effectiveness of a Community-Based Intervention for Control of Dengue Virus Vector, Ouagadougou, Burkina Faso.** *Emerg Infect Dis* 2018, **24:**1859-1867.

17. Yalwala S, Kollars JW, Kasembeli G, Barasa C, Senessie C, Kollars PG, Kollars TM: **Preliminary Report on the Reduction of Adult Mosquitoes in Housing Compounds in Western Kenya Using the ProVector Flower and Entobac Bait Pads Containing Bacillus thuringiensis israelensis With Honey Bait.** *J Med Entomol* 2016, **53:**1242-1244.

18. Nzelibe H, Albaba S: **Larvicidal Potential of *Persea americana* SeedExtract against *Aedes vittatus* Mosquito.** ***British Journal of Applied Science & Technology*** 2015, **11:**1-9.

19. Eze EA, Danga SP, Okoye FB: **Larvicidal activity of the leaf extracts of Spondias mombin Linn. (Anacardiaceae) from various solvents against malarial, dengue and filarial vector mosquitoes (Diptera: Culicidae).** *J Vector Borne Dis* 2014, **51:**300-306.

20. Goorah S, Dewkurun M, Ramchurn S: **Assessing the sustainability of individual behavior change against mosquitoes after the outbreak of a vector-borne disease in Mauritius: A case study.** *Internet Journal of Medical Update* 2014, **8**.

21. Goorah S, Russeeawon Y, Ramchurn S: **Barriers to the Usage of Topical Mosquito Repellents in Young Adults at Risk of Mosquito Bites in an Inter-epidemic Period in Mauritius.** *Asian Journal of Biomedical and Pharmaceutical Sciences* 2014, **04 (35):**18-21.

22. Kemabonta KA, Anikwe JC, Adaezeobiora IB: **Bioefficacy of Skeeter Abate and Spintor on Aanopheles gambiae and Aedes aegypti Mosquitoes from insecticide resistance areas in Lagos and Oyo States, Nigeria.** *Journal of Biology, Agriculture and Healthcare* 2013, **3**.

23. Edwin U, Nyiutaha I, Essien A, Nnamdi O, Sunday E: **Larvicidal effect of aqueous and ethanolic extracts of Senna alata on Anopheles gambiae, Culex quinquefasciatus and Aedes aegypti.** *Pakistan Journal of Pharmaceutical Sciences* 2013, **26**.

24. Khallaayoune K, Qualls WA, Revay EE, Allan SA, Arheart KL, Kravchenko VD, Xue RD, Schlein Y, Beier JC, Müller GC: **Attractive toxic sugar baits: control of mosquitoes with the low-risk active ingredient dinotefuran and potential impacts on nontarget organisms in Morocco.** *Environ Entomol* 2013, **42:**1040-1045.

25. Seidahmed OM, Siam HA, Soghaier MA, Abubakr M, Osman HA, Abd Elrhman LS, Elmagbol B, Velayudhan R: **Dengue vector control and surveillance during a major outbreak in a coastal Red Sea area in Sudan.** *East Mediterr Health J* 2012, **18:**1217-1224.

26. Mousson L, Zouache K, Arias-Goeta C, Raquin V, Mavingui P, Failloux AB: **The native Wolbachia symbionts limit transmission of dengue virus in Aedes albopictus.** *PLoS Negl Trop Dis* 2012, **6:**e1989.

27. Dube FF, Tadesse K, Birgersson G, Seyoum E, Tekie H, Ignell R, Hill SR: **Fresh, dried or smoked? Repellent properties of volatiles emitted from ethnomedicinal plant leaves against malaria and yellow fever vectors in Ethiopia.** *Malar J* 2011, **10:**375.

28. Tun-Lin W, Lenhart A, Nam VS, Rebollar-Téllez E, Morrison AC, Barbazan P, Cote M, Midega J, Sanchez F, Manrique-Saide P, et al: **Reducing costs and operational constraints of dengue vector control by targeting productive breeding places: a multi-country non-inferiority cluster randomized trial.** *Trop Med Int Health* 2009, **14:**1143-1153.

29. Seye F, Faye O, Ndiaye M, Njie E, Marie Afoutou J: **Pathogenicity of the Fungus, Aspergillus clavatus, isolated from the locust, Oedaleus senegalensis, against larvae of the mosquitoes Aedes aegypti, Anopheles gambiae and Culex quinquefasciatus.** *J Insect Sci* 2009, **9:**1-7.

30. Staikowsky F, Le Roux K, Schuffenecker I, Laurent P, Grivard P, Develay A, Michault A: **Retrospective survey of Chikungunya disease in Réunion Island hospital staff.** *Epidemiol Infect* 2008, **136:**196-206.

31. Gould LH, Osman MS, Farnon EC, Griffith KS, Godsey MS, Karch S, Mulenda B, El Kholy A, Grandesso F, de Radiguès X, et al: **An outbreak of yellow fever with concurrent chikungunya virus transmission in South Kordofan, Sudan, 2005.** *Trans R Soc Trop Med Hyg* 2008, **102:**1247-1254.

32. Dehecq J-S, Fohr G, Thiria J: **Plan de lutte contre Aedes albopictus pendant l’épidémie de chikungunyaà La Réunion en 2005-2007.** Bulletin Epidemiologique Hebdomadaire2008.

33. Dondji B, Duchon S, Diabate A, Herve JP, Corbel V, Hougard JM, Santus R, Schrevel J: **Assessment of laboratory and field assays of sunlight-induced killing of mosquito larvae by photosensitizers.** *J Med Entomol* 2005, **42:**652-656.

34. Badolo A, Ilboudo-Sanogo E, Ouédraogo AP, Costantini C: **Evaluation of the sensitivity of Aedes aegypti and Anopheles gambiae complex mosquitoes to two insect repellents: DEET and KBR 3023.** *Trop Med Int Health* 2004, **9:**330-334.

35. Bassolé IH, Guelbeogo WM, Nébié R, Costantini C, Sagnon N, Kabore ZI, Traoré SA: **Ovicidal and larvicidal activity against Aedes aegypti and Anopheles gambiae complex mosquitoes of essential oils extracted from three spontaneous plants of Burkina Faso.** *Parassitologia* 2003, **45:**23-26.

36. Konan YL, Sylla MS, Doannio JM, Traoré S: **Comparison of the effect of two excipients (karite nut butter and vaseline) on the efficacy of Cocos nucifera, Elaeis guineensis and Carapa procera oil-based repellents formulations against mosquitoes biting in Ivory Coast.** *Parasite* 2003, **10:**181-184.

37. Diallo D, Marston A, Terreaux C, Touré Y, Paulsen BS, Hostettmann K: **Screening of Malian medicinal plants for antifungal, larvicidal, molluscicidal, antioxidant and radical scavenging activities.** *Phytother Res* 2001, **15:**401-406.

38. Obeta J: **Mosquitocidal Bacillus thuringiensis from Nigerian soils.** *Insect Science and its Application* 1998, **18**.

39. Horosko S, Robert LL: **U.S. Army vector control (preventive medicine) operations during Operation Restore Hope, Somalia.** *Mil Med* 1996, **161:**577-581.

40. Karch S, Asidi N, Manzambi Z, Salaun JJ, Mouchet J: **Impact of deltamethrin-impregnated bednets on biting rates of mosquitoes in Zaire.** *J Am Mosq Control Assoc* 1995, **11:**191-194.

41. Asimeng E, Mutinga M: **A preliminary account of larvivorous fish in the Mwea Rice Irrigation System.** *Biological Control* 1993, **3**.

42. Fletcher M, Teklehaimanot A, Yemane G: **Control of mosquito larvae in the port city of Assab by an indigenous larvivorous fish, Aphanius dispar.** *Acta Trop* 1992, **52:**155-166.

43. Logan T, Linthicum K: **Evaluation of a briquet formulation of Bacillus thuringiensis var. israelensis (H-14) against Aedes spp. and Culex spp. larvae in dambos in Kenya.** *Biocontrol Science and Technology* 1992, **2**.

44. Mutinga MJ, Renapurkar DM, Wachira DW, Mutero CM, Basimike M: **Evaluation of the residual efficacy of permethrin-impregnated screens used against mosquitoes in Marigat, Baringo district, Kenya.** *Trop Med Parasitol* 1992, **43:**277-281.

45. Mwangi J, Addae-Mensah I, Muriuki G, Munavu R, Lwande W, Hassanali A: **Essential oils of Lippia species in Kenya. IV: Maize weevil (Sitophilus zeamais) repellancy and larvicidal activity.** *International Journal of Pharmacognosy* 1992, **30**.

46. Ejiofor AO, Okafor N: **Formulation of a flowable liquid concentrate of Bacillus thuringiensis serotype H-14 spores and crystals as mosquito larvicide.** *J Appl Bacteriol* 1991, **71:**202-206.

47. Logan TM, Linthicum KJ, Wagateh JN, Thande PC, Kamau CW, Roberts CR: **Pretreatment of floodwater Aedes habitats (dambos) in Kenya with a sustained-release formulation of methoprene.** *J Am Mosq Control Assoc* 1990, **6:**736-738.

48. Ejiofor A, Okafor N: **Production of mosquito larvicidal Bacillus thuringiensis serotype H-14 on raw material media from Nigeria.** *Journal of Applied Bacteriology* 1989, **67**.

49. Lindsay SW, Janneh LM: **Preliminary field trials of personal protection against mosquitoes in The Gambia using deet or permethrin in soap, compared with other methods.** *Med Vet Entomol* 1989, **3:**97-100.

50. Sabatinelli G, Majori G: **Utilisation de *Poecilia reticulata* dans la lutte contre le paludisme dans la R.F.I des Comores.** *Parassitologia* 1988, **30**.

51. Birley MH, Mutero CM, Turner IF, Chadwick PR: **The effectiveness of mosquito coils containing esbiothrin under laboratory and field conditions.** *Ann Trop Med Parasitol* 1987, **81:**163-171.

52. Nnakumusana E: **Effects of temperature on the susceptibility of *Aedes aegypti* (L.) (Diptera: Culicidae) larvae to a mosquito pathogen *Coelomomyces stegomyiae* in Uganda.** *Appl Ent Zool* 1987, **22**.

53. Nnakumusana E: **The pathogenicity of two isolates of Pythium spp. to mosquito larvae in the laboratory.** *Insect Sci Appl* 1987, **8**.

54. Jondiko I: **A mosquito larvicide in Spilanthes mauritiana.** *Phytochemistry* 1986, **25**.

55. Hougard J, Duval J, Escaffre H: **Field evaluation of larvicidal activity of a water-dispersible concentrate of Bacillus thuringiensis H-14 in a yellow fever focus in Ivory Coast.** *Cahiers ORSTOM, Serie Entomologie Medicale et Parasitologie* 1985, **23**.

56. Nnakumusana ES: **Susceptibility of mosquito larvae to Coelomomyces indicus.** *Indian J Med Res* 1985, **82:**316-320.
