# Supplementary material for: Building resilience against the growing threat of arboviruses: a scoping review of Aedes vector surveillance, control strategies and insecticide resistance in Africa
Source: Parasit Vectors. 2025 Oct 17;18:415. doi: 10.1186/s13071-025-07049-7 (PMC12535094; doi:10.1186/s13071-025-07049-7)
Supplement: Supplementary file 3 — Additional file 3: Table S3. [file 13071_2025_7049_MOESM3_ESM.docx]

| **Supplementary Table 3. Insecticide Resistance Publications** | | | | |
| --- | --- | --- | --- | --- |
| Number | Study | Publication Year | Country | Ref |
| 1 | Owusu-Asenso et al. | 2022 | Ghana | [1] |
| 2 | Abdullahi et al. | 2022 | Nigeria | [2] |
| 3 | Fagbohun et al. | 2022 | Nigeria | [3] |
| 4 | Gomard et al. | 2022 | La Réunion | [4] |
| 5 | Kampango et al. | 2022 | Tanzania | [5] |
| 6 | Kouadio et al. | 2022 | Côte d’Ivoire | [6] |
| 7 | Kwame Amlalo et al. | 2022 | Ghana | [7] |
| 8 | Lebon et al. | 2022 | La Réunion | [8] |
| 9 | Montgomery et al. | 2022 | Cameroon | [9] |
| 10 | Mukhtar et al. | 2022 | Nigeria | [10] |
| 11 | Toe et al. | 2022 | Burkina Faso | [11] |
| 12 | Yougang et al. | 2022 | Cameroon | [12] |
| 13 | Sene et al. | 2021 | Senegal | [13] |
| 14 | Djiappi-Tchamen et al. | 2021 | Cameroon | [14] |
| 15 | Konan et al. | 2021 | Côte d’Ivoire | [15] |
| 16 | Campos et al. | 2020 | Cabo Verde | [16] |
| 17 | Padonou et al. | 2020 | Benin | [17] |
| 18 | Pires et al. | 2020 | Cabo Verde | [18] |
| 19 | Ayres et al. | 2020 | Angola | [19] |
| 20 | Fagbohun et al. (a) | 2020 | Nigeria | [20] |
| 21 | Fagbohun et al. (b) | 2020 | Nigeria | [21] |
| 22 | Hondt et al. | 2020 | Cameroon | [22] |
| 23 | Kahamba et al. | 2020 | Tanzania | [23] |
| 24 | Kamgang et al. | 2020 | Republic of Congo | [24] |
| 25 | Kudom | 2020 | Ghana | [25] |
| 26 | Namountougou et al. (a) | 2020 | Burkina Faso | [26] |
| 27 | Namountougou et al. (b) | 2020 | Burkina Faso | [27] |
| 28 | Olusegun-Joseph et al. | 2020 | Nigeria | [28] |
| 29 | Yougang et al. (a) | 2020 | Cameroon | [29] |
| 30 | Yougang et al. (b) | 2020 | Cameroon | [30] |
| 31 | Sombie et al. | 2019 | Burkina Faso | [31] |
| 32 | Badolo et al. | 2019 | Burkina Faso | [32] |
| 33 | Ouattara et al. | 2019 | Burkina Faso | [33] |
| 34 | Mostafa et al. | 2019 | Egypt | [34] |
| 35 | Salgueiro et al. | 2019 | Cabo Verde | [35] |
| 36 | Bala et al. | 2018 | Sudan | [36] |
| 37 | Ukpai et al. | 2018 | Nigeria | [37] |
| 38 | Yadouleton et al. | 2018 | Benin | [38] |
| 39 | Emidi et al. | 2017 | Tanzania | [39] |
| 40 | Kamgang et al. | 2017 | Cameroon | [40] |
| 41 | Mathias et al. | 2017 | Tanzania | [41] |
| 42 | Kawada et al. | 2016 | Ghana | [42] |
| 43 | Ngoagouni et al. | 2016 | Central Africa Republic | [43] |
| 44 | Oduola et al. | 2016 | Nigeria | [44] |
| 45 | Xu et al. | 2016 | La Réunion | [45] |
| 46 | Suzuki Takashi et al. | 2016 | Ghana | [46] |
| 47 | Rocha et al. | 2015 | Cabo Verde | [47] |
| 48 | Ayorinde et al. | 2015 | Nigeria | [48] |
| 49 | Pocquet et al. | 2014 | Mayotte | [49] |
| 50 | Kemabonta et al. | 2013 | Nigeria | [50] |
| 51 | Konan et al. | 2012 | Côte d’Ivoire | [51] |
| 52 | Dia et al. | 2012 | Senegal & Cabo Verde | [52] |
| 53 | Kamgang et al. | 2011 | Cameroon & Gabon | [53] |
| 54 | Tantely et al. | 2010 | La Réunion | [54] |
| 55 | Sow et al. | 2007 | Nigeria | [55] |
| 56 | Ndams et al. | 2006 | Nigeria | [56] |
| 57 | Brengues et al. | 2003 | Côte d’Ivoire | [57] |

Table S3 shows the 57 publications that met our inclusion criteria for *Aedes* insecticide resistance testing. These studies were analyzed to determine the state of insecticide resistance monitoring of *Aedes* vectors in Africa, including bioassay methodologies, insecticides, concentrations and mosquito species used, and major mechanisms of insecticide resistance.

**References**

1. Owusu-Asenso CM, Mingle JAA, Weetman D, Afrane YA: **Spatiotemporal distribution and insecticide resistance status of Aedes aegypti in Ghana.** *Parasit Vectors* 2022, **15:**61.

2. Abdullahi Y, M., Fana S, Bandiya H, Batagarawa U, Zulkarnain M, Uthman H: **Susceptibility status of Aedes aegypti to pyrethroids in Usmanu Danfodiyo University main campus, Sokoto, Nigeria.** *International Journal of Mosquito Research* 2022, **9**.

3. Fagbohun IK, Oyeniyi TA, Idowu ET, Nwanya O, Okonkwo F, Adesalu KO, Jimoh RT, Oladosu Y, Otubanjo OA, Adeogun AO: **Detection and Co-occurrence of kdr (F1534C and S989P) Mutations in Multiple Insecticides Resistant Aedes aegypti (Diptera: Culicidae) in Nigeria.** *J Med Entomol* 2022, **59:**1741-1748.

4. Gomard Y, Alout H, Lebon C, Latreille A, Benlali A, Mavingui P, Tortosa P, Atyame C: **Fitness costs associated with a GABA receptor mutation conferring dieldrin resistance in Aedes albopictus.** *Heredity (Edinb)* 2022, **129:**273-280.

5. Kampango A, Hocke EF, Hansson H, Furu P, Haji KA, David JP, Konradsen F, Saleh F, Weldon CW, Schiøler KL, Alifrangis M: **High DDT resistance without apparent association to kdr and Glutathione-S-transferase (GST) gene mutations in Aedes aegypti population at hotel compounds in Zanzibar.** *PLoS Negl Trop Dis* 2022, **16:**e0010355.

6. Kouadio A, Guindo-Coulibaly N, Adja A, Kpan S, Assouho K, Zoh D, Yapi A, Azongnibo K, Sadia-Kacou A: **High density in dry season and resistance to pyrethroids of Aedes aegypti populations in Bouake, Cote d'Ivoire.** *International Journal of Tropical Insect Science* 2022.

7. Kwame Amlalo G, Akorli J, Etornam Akyea-Bobi N, Sowa Akporh S, Aqua-Baidoo D, Opoku M, Frempong K, Pi-Bansa S, Boakye HA, Joannides J, et al: **Evidence of High Frequencies of Insecticide Resistance Mutations in Aedes aegypti (Culicidae) Mosquitoes in Urban Accra, Ghana: Implications for Insecticide-based Vector Control of Aedes-borne Arboviral Diseases.** *J Med Entomol* 2022, **59:**2090-2101.

8. Lebon C, Alout H, Zafihita S, Dehecq JS, Weill M, Tortosa P, Atyame C: **Spatio-Temporal Dynamics of a Dieldrin Resistance Gene in Aedes albopictus and Culex quinquefasciatus Populations From Reunion Island.** *J Insect Sci* 2022, **22**.

9. Montgomery M, Harwood JF, Yougang AP, Wilson-Bahun TA, Tedjou AN, Keumeni CR, Kilpatrick AM, Wondji CS, Kamgang B: **Spatial distribution of insecticide resistant populations of Aedes aegypti and Ae. albopictus and first detection of V410L mutation in Ae. aegypti from Cameroon.** *Infect Dis Poverty* 2022, **11:**90.

10. Mukhtar MM, Ibrahim SS: **Temporal Evaluation of Insecticide Resistance in Populations of the Major Arboviral Vector.** *Insects* 2022, **13**.

11. Toé HK, Zongo S, Guelbeogo MW, Kamgang B, Viana M, Tapsoba M, Sanou A, Traoré A, McCall PJ, Sagnon N: **Multiple insecticide resistance and first evidence of V410L kdr mutation in Aedes (Stegomyia) aegypti (Linnaeus) from Burkina Faso.** *Med Vet Entomol* 2022, **36:**309-319.

12. Yougang AP, Keumeni CR, Wilson-Bahun TA, Tedjou AN, Njiokou F, Wondji C, Kamgang B: **Spatial distribution and insecticide resistance profile of Aedes aegypti and Aedes albopictus in Douala, the most important city of Cameroon.** *PLoS One* 2022, **17:**e0278779.

13. Sene NM, Mavridis K, Ndiaye EH, Diagne CT, Gaye A, Ngom EHM, Ba Y, Diallo D, Vontas J, Dia I, Diallo M: **Insecticide resistance status and mechanisms in Aedes aegypti populations from Senegal.** *PLoS Negl Trop Dis* 2021, **15:**e0009393.

14. Djiappi-Tchamen B, Nana-Ndjangwo MS, Mavridis K, Talipouo A, Nchoutpouen E, Makoudjou I, Bamou R, Mayi AMP, Awono-Ambene P, Tchuinkam T, et al: **Analyses of Insecticide Resistance Genes in.** *Genes (Basel)* 2021, **12**.

15. Konan LY, Oumbouke WA, Silué UG, Coulibaly IZ, Ziogba JT, N'Guessan RK, Coulibaly D, Bénié JBV, Lenhart A: **Insecticide Resistance Patterns and Mechanisms in Aedes aegypti (Diptera: Culicidae) Populations Across Abidjan, Côte d'Ivoire Reveal Emergent Pyrethroid Resistance.** *J Med Entomol* 2021, **58:**1808-1816.

16. Campos KB, Martins AJ, Rodovalho CM, Bellinato DF, Dias LDS, Macoris MLDG, Andrighetti MTM, Lima JBP, Obara MT: **Assessment of the susceptibility status of Aedes aegypti (Diptera: Culicidae) populations to pyriproxyfen and malathion in a nation-wide monitoring of insecticide resistance performed in Brazil from 2017 to 2018.** *Parasit Vectors* 2020, **13:**531.

17. Padonou G, Osse R, Salako A, Aikpon R, Sovi A, Kpanou C, Sagbohan H, Akadiri Y, Lamine B, Akogbeto M: **Entomological assessment of the risk of dengue outbreak in Abomey-Calavi Commune, Benin.** *Tropical Medicine and Health* 2020.

18. Pires S, Alves J, Dia I, Gómez LF: **Susceptibility of mosquito vectors of the city of Praia, Cabo Verde, to Temephos and Bacillus thuringiensis var israelensis.** *PLoS One* 2020, **15:**e0234242.

19. Ayres CFJ, Seixas G, Borrego S, Marques C, Monteiro I, Marques CS, Gouveia B, Leal S, Troco AD, Fortes F, et al: **The V410L knockdown resistance mutation occurs in island and continental populations of Aedes aegypti in West and Central Africa.** *PLoS Negl Trop Dis* 2020, **14:**e0008216.

20. Fagbohun I, Idowu E, Olakiigbe A, Oyeniyi A, Otubanjo O, Awolola T: **Metabolic resistance mechanism in Aedes aegypti from Lagos State, Nigeria.** *Journal of Basic and Applied Zoology* 2020, **81**.

21. Fagbohun I, Idowu E, Otubanjo O, Awolola T: **Susceptibility status of mosquitoes (Diptera: Culicidae) to malathion in Lagos, Nigeria.** *Animal Research International* 2020, **17**.

22. Hondt O, Akono P, Hiol J, Edou D, Tonga C, Dadji G, Kekeunou S: **Competitive adaptation of Aedes albopictus, Skuse 1894 in the presence of Aedes aegypti Linne 1862 in temporary larvae breeding sites and in the context of pyrethroids resistance in Douala (Cameroon).** *Bulletin de la Societe de Pathologie Exotique* 2020, **113**.

23. Kahamba NF, Limwagu AJ, Mapua SA, Msugupakulya BJ, Msaky DS, Kaindoa EW, Ngowo HS, Okumu FO: **Habitat characteristics and insecticide susceptibility of Aedes aegypti in the Ifakara area, south-eastern Tanzania.** *Parasit Vectors* 2020, **13:**53.

24. Kamgang B, Wilson-Bahun TA, Yougang AP, Lenga A, Wondji CS: **Contrasting resistance patterns to type I and II pyrethroids in two major arbovirus vectors Aedes aegypti and Aedes albopictus in the Republic of the Congo, Central Africa.** *Infect Dis Poverty* 2020, **9:**23.

25. Kudom AA: **Entomological surveillance to assess potential outbreak of Aedes-borne arboviruses and insecticide resistance status of Aedes aegypti from Cape Coast, Ghana.** *Acta Trop* 2020, **202:**105257.

26. Namountougou M, Soma DD, Balboné M, Kaboré DA, Kientega M, Hien A, Coulibaly A, Ouattara PE, Meda BG, Drabo S, et al: **Monitoring Insecticide Susceptibility in.** *Trop Med Infect Dis* 2020, **5**.

27. Namountougou M, Soma D, Kabore D, N'do S, Kientega M, Sawadogo J, Kagone T, Kania D, Sanou R, Sangare I, et al: **Characterisation of the breeding sites and insecticide resistance of Aedes aegypti population in the city of Bobo-Dioulaso, Burkina Faso.** *African Entomology* 2020, **28**.

28. Olusegun-Joseph T, Oboh M, Awoniyi A, Adebowale A, Agbaso M, Fagbohun I: **Efficacy of piperonyl butoxide (PBO) synergist on pyrethroid and dichlorodiphenyl trichloroethane (DDT) resistant mosquitoes in Lekki, Lagos State, Nigeria.** *Animal Research International* 2020, **17:**3821-3828.

29. Yougang AP, Kamgang B, Bahun TAW, Tedjou AN, Nguiffo-Nguete D, Njiokou F, Wondji CS: **First detection of F1534C knockdown resistance mutation in Aedes aegypti (Diptera: Culicidae) from Cameroon.** *Infect Dis Poverty* 2020, **9:**152.

30. Yougang AP, Kamgang B, Tedjou AN, Wilson-Bahun TA, Njiokou F, Wondji CS: **Nationwide profiling of insecticide resistance in Aedes albopictus (Diptera: Culicidae) in Cameroon.** *PLoS One* 2020, **15:**e0234572.

31. Sombié A, Saiki E, Yaméogo F, Sakurai T, Shirozu T, Fukumoto S, Sanon A, Weetman D, McCall PJ, Kanuka H, Badolo A: **High frequencies of F1534C and V1016I.** *Trop Med Health* 2019, **47:**2.

32. Badolo A, Sombié A, Pignatelli PM, Sanon A, Yaméogo F, Wangrawa DW, Kanuka H, McCall PJ, Weetman D: **Insecticide resistance levels and mechanisms in Aedes aegypti populations in and around Ouagadougou, Burkina Faso.** *PLoS Negl Trop Dis* 2019, **13:**e0007439.

33. Ouattara LPE, Sangaré I, Namountougou M, Hien A, Ouari A, Soma DD, Kassié D, Diabaté A, Gnankiné O, Bonnet E, et al: **Surveys of Arboviruses Vectors in Four Cities Stretching Along a Railway Transect of Burkina Faso: Risk Transmission and Insecticide Susceptibility Status of Potential Vectors.** *Front Vet Sci* 2019, **6:**140.

34. Mostafa A, Rashed M, Aly N, Hasan A, Mikhail M: **Entomological Surveillance of Aedes aegypti and Arboviruses Outbreak of Dengue Fever In The Red Sea Governorate, Egypt.** *Journal of the Egyptian Society of Parasitology* 2019, **49**.

35. Salgueiro P, Serrano C, Gomes B, Alves J, Sousa CA, Abecasis A, Pinto J: **Phylogeography and invasion history of.** *Evol Appl* 2019, **12:**1797-1811.

36. Bala A, Abakar A, Khair M, Mohammed M: **First report on insecticides susceptibility of restricted foci of Aedes aegypti, a viruses' vector, in Gezira state, Sudan.** *International Journal of Mosquito Research* 2018, **5**.

37. Ukpai O, Ekedo C: **Insecticide susceptibility status of Aedes aegypti in Umudike, Ikwuano LGA Abia State, Nigeria.** *Animal Research International* 2018, **15**.

38. Yadouleton C, Tchibozo C, Azondekon R, Agbanrin R, Ahissou F, Houndeton A, Sidick A, Gbaguidi F, Akogbeto M: **Pyrethroid resistance in Aedes aegypti populations in southern Benin, West Africa.** *International Journal of Mosquito Research* 2018, **5**.

39. Emidi B, Kisinza W, Kaaya R, Malima R, Mosha F: **Insecticide susceptibility status of human biting mosquitoes in Muheza, Tanzania.** *Tanzania Journal of Health Research* 2017, **19**.

40. Kamgang B, Yougang AP, Tchoupo M, Riveron JM, Wondji C: **Temporal distribution and insecticide resistance profile of two major arbovirus vectors Aedes aegypti and Aedes albopictus in Yaoundé, the capital city of Cameroon.** *Parasit Vectors* 2017, **10:**469.

41. Mathias L, Baraka V, Philbert A, Innocent E, Francis F, Nkwengulila G, Kweka EJ: **Habitat productivity and pyrethroid susceptibility status of Aedes aegypti mosquitoes in Dar es Salaam, Tanzania.** *Infect Dis Poverty* 2017, **6:**102.

42. Kawada H, Higa Y, Futami K, Muranami Y, Kawashima E, Osei JH, Sakyi KY, Dadzie S, de Souza DK, Appawu M, et al: **Discovery of Point Mutations in the Voltage-Gated Sodium Channel from African Aedes aegypti Populations: Potential Phylogenetic Reasons for Gene Introgression.** *PLoS Negl Trop Dis* 2016, **10:**e0004780.

43. Ngoagouni C, Kamgang B, Brengues C, Yahouedo G, Paupy C, Nakouné E, Kazanji M, Chandre F: **Susceptibility profile and metabolic mechanisms involved in Aedes aegypti and Aedes albopictus resistant to DDT and deltamethrin in the Central African Republic.** *Parasit Vectors* 2016, **9:**599.

44. Oduola A, Obembe A, Adelaja O, Ande A: **Surveillance and insecticide susceptibility status of Culicine mosquitoes in selected communities utilizing long-lasting insecticidal nets in Kwara State, Nigeria.** *Animal Research International* 2016, **13**.

45. Xu J, Bonizzoni M, Zhong D, Zhou G, Cai S, Li Y, Wang X, Lo E, Lee R, Sheen R, et al: **Multi-country Survey Revealed Prevalent and Novel F1534S Mutation in Voltage-Gated Sodium Channel (VGSC) Gene in Aedes albopictus.** *PLoS Negl Trop Dis* 2016, **10:**e0004696.

46. Suzuki T, Osei JH, Sasaki A, Adimazoya M, Appawu M, Boakye D, Ohta N, Dadzie S: **Risk of transmission of viral haemorrhagic fevers and the insecticide susceptibilitystatus of.** *Ghana Med J* 2016, **50:**136-141.

47. Rocha HDR, Paiva MHS, Silva NM, de Araújo AP, Camacho DDRD, Moura AJFD, Gómez LF, Ayres CFJ, Santos MAVM: **Susceptibility profile of Aedes aegypti from Santiago Island, Cabo Verde, to insecticides.** *Acta Trop* 2015, **152:**66-73.

48. Ayorinde A, Oboh B, Oduola A, Otubanjo O: **The Insecticide Susceptibility Status of Aedes aegypti (Diptera: Culicidae) in Farm and Nonfarm Sites of Lagos State, Nigeria.** *J Insect Sci* 2015, **15**.

49. Pocquet N, Darriet F, Zumbo B, Milesi P, Thiria J, Bernard V, Toty C, Labbé P, Chandre F: **Insecticide resistance in disease vectors from Mayotte: an opportunity for integrated vector management.** *Parasit Vectors* 2014, **7:**299.

50. Kemabonta KA, Anikwe JC, Adaezeobiora IB: **Bioefficacy of Skeeter Abate and Spintor on Aanopheles gambiae and Aedes aegypti Mosquitoes from insecticide resistance areas in Lagos and Oyo States, Nigeria.** *Journal of Biology, Agriculture and Healthcare* 2013, **3**.

51. Konan LY, Coulibaly IZ, Kone BA, Ziogba JC, Diallo A, Ekra DK, Traoré KS, Doannio MC, Paul OK: **Aedes aegypti susceptibility to insecticide from Abidjan City, Cote D'ivoire.** *Vector Borne Zoonotic Dis* 2012, **12:**325-329.

52. Dia I, Diagne CT, Ba Y, Diallo D, Konate L, Diallo M: **Insecticide susceptibility of Aedes aegypti populations from Senegal and Cape Verde Archipelago.** *Parasit Vectors* 2012, **5:**238.

53. Kamgang B, Marcombe S, Chandre F, Nchoutpouen E, Nwane P, Etang J, Corbel V, Paupy C: **Insecticide susceptibility of Aedes aegypti and Aedes albopictus in Central Africa.** *Parasit Vectors* 2011, **4:**79.

54. Tantely ML, Tortosa P, Alout H, Berticat C, Berthomieu A, Rutee A, Dehecq JS, Makoundou P, Labbé P, Pasteur N, Weill M: **Insecticide resistance in Culex pipiens quinquefasciatus and Aedes albopictus mosquitoes from La Réunion Island.** *Insect Biochem Mol Biol* 2010, **40:**317-324.

55. Sow G, Ndams I, Kogi E, Tukur Z, Adamu H: **Efficacy of permethrin insecticide tested against populations of Anopheles and Aedes from different larval habitats in southern Guinea Savanna, Nigeria.** *Science World Journal* 2007, **2**.

56. Ndams I, Laila K, Tukur Z: **Susceptibility of some species of mosquitoes to permethrin pyrethroid in Zaria Nigeria.** *Science World Journal* 2006, **1**.

57. Brengues C, Hawkes NJ, Chandre F, McCarroll L, Duchon S, Guillet P, Manguin S, Morgan JC, Hemingway J: **Pyrethroid and DDT cross-resistance in Aedes aegypti is correlated with novel mutations in the voltage-gated sodium channel gene.** *Med Vet Entomol* 2003, **17:**87-94.
